# Social movements and the struggle for shelter: A case study of eThekwini (Durban)^☆^

**DOI:** 10.1016/j.progress.2012.12.001

**Published:** 2013-08

**Authors:** Diana Mitlin, Jan Mogaladi

**Affiliations:** a University of Manchester, United Kingdom; b University of the Western Cape, South Africa

**Keywords:** Housing policy, eThekwini/Durban, South Africa, Low-income shelter, Urban social movements

## Abstract

The needs of informal settlement dwellers across towns and cities in the global South are acute. While much emphasis has been placed on income poverty, for urban dwellers affordable access to improved secured accommodation and basic services such as water and sanitation is essential for well-being. In part due to the lack of such access, urban citizens organize in multiple ways to address these needs and to press for state investment and redistribution. South Africa, despite relatively high levels of income and a state with redistributive capacity, is no exception. Indeed, this country is particularly interesting for those seeking to understand effective approaches to addressing housing need because of the efforts that have been made and the resultant scale of state investment in the housing sector. This paper draws on the experiences of urban social movement organizations in South Africa to understand better how shelter needs can be addressed. We reflect on their goals, strategies and activities and explore what this means for themes discussed within the social movement literature. The research has a specific focus on the city of eThekwini/Durban primarily because of the scale of social movement activities there.

The paper describes the core concerns with respect to shelter as elaborated by social movement activists, staff of other civil society agencies and officials. While there is a broad consensus about the shelter problems, there is less agreement about solutions. The discussion summarises the shelter objectives and core strategies used by the social movement activists, and considers the success they have had. This includes reviewing their understanding of what success is and how success is achieved. Movement organisations have been able to secure access to the housing subsidy scheme for their members. However, their progress has been limited. The paper concludes that there is a primary focus on what is offered through state programmes and policies despite evident shortcomings. While there is an awareness of the misfit between available programmes, the needs of movement members and more general shelter needs, no fundamental challenge is being made to the dominant approach. A second finding is that while movement members and leaders have an explicit focus on material improvements, underlying their motivations and some of their strategies and actions is the more fundamental goal to be treated as equal citizens. In this dimension, movement organizations seek both recognition of their citizenship as well as the redistribution of state resources. Third, we argue that while social movement progress is related to the political opportunity structures, it also depends on the ability of movement organizations to manage the emerging opportunities, particularly their relationships with the state, professional support organizations and other movement organizations. Relations shift between contention and collaboration to improve negotiating positions, secure access to shelter and achieve greater political inclusion.

## Highlights

• Housing policy in South Africa has not successfully addressed the scale of housing needs despite considerable achievements.
• Low-income households are frustrated and participate in urban social movements to advance interests and address needs.
• Movement activists and government staff agree about the limited outcomes of housing policy and the scale of housing needs.
• Movements and state agencies use many strategies to improve their political position and strengthen their negotiations.
• Political opportunity processes/structures are not fixed and movements influence such opportunities and constraints.

## 1 Introduction

This paper examines the challenge of housing provision in South Africa and the perspectives and contribution of social movements to addressing housing need. South Africa has made a significant investment in housing since 1994 and the government has financed the construction of more than 2 million houses. Despite this the housing backlog is increasing and millions remain in housing need, living in informal settlements or renting rooms in formal areas.

There is a long tradition of social movement activism related to collective consumption in the towns and cities of the North and South. This involves securing access to essential goods and services including secure tenure, access to water, sanitation and other basic services, and housing. Such goods and services are generally supplied to groups of low-income citizens (rather than individuals) and are of particular importance in urban areas.^1^ The historical significance of South Africa's social movement organisations and their struggle against apartheid is widely acknowledged. What has become apparent in recent years is the rising frustration in some low-income settlements as residents believe they have had to wait too long for improvements.^2^ Nevertheless a generous subsidy means that the primary route for comprehensive improvements in access to tenure and services continues to be seen as home ownership and securing inclusion within the government programme.

Drawing on research in the city of Durban (now renamed eThekwini), the paper examines housing provision in the city and the contributions of two active social movement organisations to addressing housing need, the Federation of the Urban Poor (FedUP) and Abahlali baseMjondolo (AbM). The research findings draw out a number of their perspectives and experiences including their views on current policies and programmes, how organised citizens have sought to participate in the design and implementation of housing programmes, and how different groups within government have responded to social movement protest and engagement. The findings help us to understand what has been achieved by social movement organisations, why it has been achieved, and what remains to be done. The findings also enable us to reflect on what these experiences mean for broader theories that are used to explain social movement achievements. Section 2 elaborates on the methodology used in the study and the context in which the research took place.

Sections 3 and 4 summarise what is already known. Section 3 focuses on government intervention in low-income housing in South Africa and explores the scale of need, the policy response and experiences with associated housing programmes, most notably the capital subsidy programme developed after 1994. It reviews the main concerns expressed by civil society organisations both through their critical discourse on the policy, and through the response of organised social movement organisations and families who have sought to address their own needs. Section 4 discusses the literature on social movements and its contribution to our understanding of collective citizen action to address shelter needs in South Africa. The strategies of social movements in the global North (particularly the United States and Europe) have been extensively theorised but there has been relatively little written about the work of such movements in the global South (Africa, Asia and Latin America). A number of recent studies have considered social movement activities in South Africa using ideas drawn from this literature and highlighting differences in strategy as movements seek to engage the state to advance their needs and interests.

Section 5 presents a brief history of the development of low-income housing provision in Durban/eThekwini and summarises views of interviewees on housing need and housing policy in the city. As elaborated below, in the analysis of the housing challenge, there is a considerable overlap in perspectives across both government and civil society. This section also includes a description of the social movements whose work is discussed in this paper. Section 6 then describes and analyses the strategies used by the two major social movement organisations. Following a description of the experiences of social movement activism, we report on the views and perspectives of interviewees both from civil society organisations and government organisations in respect of the success achieved by the strategies of collaboration and confrontation. Not surprisingly, success in terms of influencing policy and securing resources reflects the ability of the shelter movement to understand and influence political relations. Some success has been achieved but major difficulties remain and movement organisations struggle to work within state bureaucracy and face ambivalence from local councillors.

Section 7 concludes and considers what this research suggests for our understanding of the dynamic relationship between social movement organisations and activists and the state. The discussion explores the complexity of relations, and the interaction between particular strategies to advance claims, negotiate entitlements, and secure inclusion. It considers the importance of movement organisations having the ability to manage relationships both with the state and other civil society organisations to secure their needs and advance their interests. The findings emphasise the need to recognise that rather than simply being contentious, social movement organisations manage their relations with the state seeking to advance their claims, challenge inequalities in access to government institutions and gain a reputation for being legitimate agencies both in terms of resource redistribution and inclusion in decision making.

Despite an emphasis on material needs and state redistribution, the objectives of movement activists are wider than improved housing, and movement leaders are also concerned that they are treated with greater dignity and respect, and have possibilities to improve their livelihoods and secure access to a range of other services. While the government's housing programme has addressed the needs of some, its scale remains inadequate, and a lack of access compounds social isolation and political exclusion. Substantial housing needs remain. However, there is no agreement on an alternative approach and, more significantly, relatively little discussion about such options. Both the state and movement activities are orientated to making the existing system work despite its demonstrated short-comings, and little attention is given to new and alternative approaches to addressing housing needs and inequalities.

## 2 Research design

Relatively little attention has been paid by professional researchers and development commentators to establishing the overall significance of social movements to low-income and disadvantaged people; and understanding the strategic choices facing such grassroots movements, their preferred strategies and the effectiveness of these strategies in given political and economic contexts. The research reported on here is part of a wider study exploring these knowledge gaps with reference to social movement activity in Peru and South Africa. The research project began in 2007 with the mapping processes in both countries to assess the scale and intensity of movement activities in fields broadly related to poverty and poverty reduction. An interest in movement-state interactions around poverty meant the researchers sought countries with relatively consolidated state bureaucracies and significant movement activity, and established local research organisations. This led to South Africa and Peru.

The research involved two principal phases. Phase one involved a ‘mapping’ of movements, tracing the main domains of movement activity, the main organisations involved in this activity, and the interactions among these domains and organisations. The mapping asked if and how movements thought about and addressed the question of poverty. This phase of research involved interviews with established movement leaders, movement intellectuals and key informants as well as a review published and secondary material (45 interviews in South Africa, 56 in Peru). The mapping papers were discussed in each country at workshops with movement leaders, movement organisations, researchers and activists (one workshop in Lima, three in South African cities). This approach permitted a view of movements with a broad reach over space and time, and made it easier to identify and trace relationships within and among movements. Likewise the approach offered a clearer sense of patterns in the ways that different movements interact with the state, political parties and NGOs, and permitted comparison among different movements’ discourses on poverty and strategies assumed for engaging with the state on issues defined as poverty reduction by government.

The second phase involved six in-depth case studies, three in each country. The unit of analysis was the movement, though in order to ground analysis and interviews, the cases focused on particular histories, organisations and sites of movement activity. The cases were selected on the basis of suggestions at the workshops discussing the mapping papers. Case study research involved in-depth (sometimes repeat) interviews with movement leaders, members, observers, activists, relevant government officials and key informants. This was combined with analysis of secondary material. Findings were discussed in workshops with movement leaders, activists, researchers and others in both countries, and with researchers in the UK.

### 2.1 Phase one in South Africa

The mapping of social movements in South Africa highlighted several overlapping themes. In total, 36 movement organisations and their associated activities were identified and considered. In terms of the longevity and scale of movement organisations, 18 had been founded since 2000, and a further 11 were founded between 1994 and 2000. Of the movements studied, shelter (housing, land and basic services) formed the most significant focus of movement activities in terms of the numbers of organisations interviewed in the course of the mapping study. Other movements were the human rights movement, the labour movement, the feminist and environmental movements. Within the shelter movement, there were different orientations (for example, to housing or to basic services). There were also evident overlaps between the movements. For example, although there was an apparent lack of attention to the feminist cause, gender issues were taken up by shelter movement organisations (and others).

Interviewees and workshop participants in the first phase suggested considerable complexity in the relationships between social movements and the state. Some argued that state activity to address shelter need supported claim-making in the area, and may help to explain the scale of activities. At the same time, evident contradictions in state policies, for example, in the stated commitment to participation but limitations in practice, and in the emphasis on the right to water but only partial access, create tensions and may also be relevant in explaining the scale of activity. What also emerged from the first phase was a strong consciousness about movement organisations as a potential site of political opposition. Some of the most explicitly oppositional movement organisations included in the first phase are led by individuals expelled from the ANC who have chosen to locate themselves within movement organisations. Within social movement organisations, they return to the historical practice of the street protests that challenged and defeated apartheid. Their objective of challenging the ANC does not appear to be shared by most other movement organisations, or by all their own members. However, it was agreed by many interviewees that one consequence was that the state viewed some movement organisations as antagonistic and hence sought to de-legitimise them, while engaging with others.

### 2.2 From phase one to phase two

Phase one led us to conclude that social movements are best understood as networks of actors rather than individual organisations, including membership-based organisations, NGOs, and activists. (Though we noted that there are sometimes disagreements as to who is actually part of particular movements.) Boundaries among movements are porous, and organisations and individuals may participate in more than one movement. Some movements grow out of others.

Concerns emphasised by movements and their participating movement organisations differed across the two countries. In South Africa, areas of collective consumption such as urban land, housing and basic services have drawn most activism. This conclusion is consistent with Mohanty, Thompson, and Coelho (2011, 16) and a study of social mobilisation and state interaction in Brazil, India and South Africa. In Peru, issues of rights and livelihood are more prominent concerns, and reflected in mobilisation around ethnicity, extractive industry and human rights. This may reflect the different national political economy contexts. The South African government has prioritised collective consumption, opening possibilities for social movement engagement. The Peruvian government has prioritised a growth model as its main instrument of poverty reduction, inducing contention around the legitimacy and viability of this model. In both countries, movements rarely see ‘being poor’ as their main identity. Their struggles respond to being denied their rights, excluded or treated unjustly and inequitably. Experiences illustrate the diversity of strategies and tactics movements use.

Three of the movements selected for detailed study focussed on the needs of those living in low-income urban settlements and two of these were in South Africa (the other South African study looked at basic services in Cape Town). In eThekwini/Durban, as elaborated below, two substantive movement organisations provide a contrasting set of histories, experiences, relationships and strategies used to access to state housing finance and improve tenure security. Research themes include the strategies used by movement organisations seeking enhanced inclusion and recognition, and the redistribution, transfer or generation of material benefits. This case study of shelter movements in eThekwini was completed in February to November 2009. Initial contacts in eThekwini were made during phase one with interviewing among activists and key informants, and a report back in the city. These contacts plus existing relations formed the starting point for the interviews in phase two. Snowballing techniques were used to identify further interviewees with consultations and triangulation with three groups (council, professionals and movements activists) being used. Most interviews were in English and most took place in the offices or meeting rooms of council and civil society activists respectively. Interviews were taped and varied between one to two hours in length. In total, 25 key informants were interviewed of which six were members and leaders of community organisations, four were NGO staff members, seven were local authority and provincial officials, and six were commentators with experiences of movement activities. Government officials included those at the provincial and city level responsible for drafting and for implementing policy. Two of those interviewed were councillors. In addition, three focus groups were also held with social movement activists in their local neighbourhoods. In this paper, we have anonymised comments except for those from the leaders of the community organisations.

Following completion of a draft report which was circulated to interviewees, the initial research findings were presented and discussed at a day-long workshop in the city in August 2009. All interviewees were invited to attend and the final audience included about one third of the original interviewees together with a similar number of interested individuals from the same movement organisations and council. Some subsequent follow-up interviews with both council staff and movement leaders took place between September and November 2009.

## 3 A response to housing need: housing policy in South Africa

This section introduces the housing policy and subsequent programming that emerged in South Africa following democratisation in 1994. It explains the context prevailing prior to 1994, the response of the state and the progress that has been achieved.

### 3.1 The context and policy response

One of the most powerful ways in which the apartheid regime in South Africa confirmed its dominance and secured associated benefits was through the spatial segregation that it enforced over the territory. In their daily experience, African, Indian and coloured South Africans were forced to comply with laws that restricted their movement, and to accept the consequences of such restrictions for their livelihoods. Many remained in rural areas. Some were legally entitled to be in urban areas because of their employment status, while others broke the law and migrated to towns in search of employment. They located in informal settlements around the black township areas or illegally rented backyard shacks within these areas. Their presence was often challenged with particularly brutal and extensive evictions during the 1960s and 1970s.

Despite considerable repression, there was also pressure for political change. By the mid-1980s, there was a critical mass of political protest related in part to housing and residency policies; this was linked to the rise of the civic associations and activism in many towns and cities. Seekings (2000, 833) explains how, by 1986, the state's authority in black township areas had become limited and “anti-apartheid activists assumed many of the administration, everyday policing and judicial roles”. At the advent of democracy in 1994, migration to urban areas combined with significant levels of poverty to result in a massive and complex housing challenge in urban areas with an estimated housing backlog of 1.5–2 million households (Statistics South Africa, 2001).

Due to this history, housing has both a material and symbolic dimension in South Africa. In terms of its physical dimensions, housing provides safety and security for its occupants as well as helping to ensure access to basic services and, ideally, offering access to livelihoods. However, housing also has other powerful associations. Tenure security through the legal right to occupy a brick house in an urban area offers a confirmation of citizenship with associated rights and entitlements. The importance of history resonates through discussions about housing and access to housing; see, for example, Cherry, Jones, and Seekings (2000), Miraftab (2003), Skuse and Cousins (2007), Pithouse (2008a), and Mohanty et al. (2011). Interviewees frequently made references to housing struggles prior to 1994.

Following the ANC government taking up office in 1994, the right to housing was introduced into the constitution and provision of housing was declared to be a priority with a target of one million dwellings within five years. To achieve this aim, the government introduced a capital subsidy programme for land purchase, infrastructure and housing development. While the focus on housing reflected political priorities and social needs, the specific strategy of a capital subsidy for addressing housing need emerged from the business representatives and consultants who dominated the multi-stakeholder National Housing Forum between 1992 and 1994 (Baumann, 2003, 6; Huchzermeyer, 2003a, 604; Gilbert, 2002). Gilbert (2002, 1923) notes that nine of the 16 Forum's founding members represented business or pro-business interests. Irrespective of the interests favoured, the idea of a capacity subsidy appears to have appealed to an ANC government anxious to put in place a programme offering housing at scale. The capital subsidy promised the government a win-win-win option, simultaneously addressing the needs of low-income households without adequate housing, providing reassurance to a struggling construction sector, and catalysing a lead sector for economic regeneration.

The South African housing subsidy programme^3^ has been amended over the years since 1995 but remains broadly the same in structure. It offers financial support through a range of sub-programmes^4^:

• *Project linked and individual subsidies* provide finance for ownership tenure for houses built either by developers or by the beneficiaries themselves through a particular sub-programme known as the “People's Housing Process”. This programme is for those without any present access to formal housing;
• *Consolidation* (or “top-up”) subsidies provide a grant to improve shelter developed under subsidy dispensations prior to 1994. This programme is for those who benefited from previous programmes and accessed serviced sites but who need additional finance to improve the standard of their dwelling;
• *Institutional subsidies*, providing a grant to a housing association or landlord who provides housing for rent to eligible beneficiaries; and
• *Relocation assistance* offered to assist borrowers in mortgage arrears to relocate to more affordable housing.

When the programme was first introduced, project-linked and individual subsidies provided a maximum of R15,000 (about US$2150^5^) per household for those with monthly incomes below R1500 (Porteous, 2005).^6^ To be eligible, households had to include adults with legal South African residency, meet specified income criteria (under R3500 or $500 a month for major beneficiaries of the programme), have not previously received state housing assistance and have dependents. While the subsidy is, in theory, accessible to individual households, it has proved to be very difficult for single households to purchase a dwelling with their subsidy monies. Families in housing need access subsidy finance as part of a project-linked programme with the funds released to a developer (either private contractors and/or municipalities with the former being particularly important between 1994 and 2000). Baumann (2003, 9) notes that over 90% of subsidies have been allocated via the project-linked route with perhaps another 3% allocated to the People's Housing Process (PHP). The PHP is a community-led programme within the project-linked subsidy and is described below.

A further assumption made during programme design was that banks would subsequently offer top-up loans to finance improvements. However in practice this has not been forthcoming (Porteous, 2005; Rust, 2006). The failure to develop a credit-linked subsidy option has meant that most subsidised housing delivery has been for dwellings that are only financed through the subsidy. Early in the programme's implementation there were complaints about the size and quality of housing (see Zack & Charlton, 2003 and below). As a result of such complaints, a minimum size unit of 30 m^2^ was introduced in the late 1990s and this has since been expanded to 40 m^2^ (Rust, 2006). A savings requirement (of R2479) was introduced for those entitled to the subsidy but with slightly higher incomes (Baumann, 2003; Huchzermeyer, 2003a). A further amendment, introduced in response to the concerns that some houses were being abandoned (see below), was that houses cannot be sold for eight years (Porteous, 2005, 35), reduced to five years in 2005 (Lemanski, 2011).

Rental housing formed 31% of the total housing stock in 1999 with 69% of this rental housing being located in urban areas (Department of Housing, 2003, 8). Recognising the significance of rental housing, the government developed a social rental housing option within its housing programme. However, between 1996 and December 2005, the National Department of Housing recorded the delivery of less than 35,000 units across the country. Very little new development has happened in subsequent years.

### 3.2 Progress in subsidy delivery

By 2003, one senior academic commentator, Huchzermeyer (2003b, 212) was arguing that South Africa's housing policy was recognised as being successful with “82% of the South African housing budget (currently 2.6% of the national budget) being spent on once-off supply-side capital subsidies.” By March 2009, 2.8 million subsidised housing units had been provided or were being constructed.^7^ However, neither the scale nor subsequent modifications to the programme has resulted in unambiguous success. In 1996, 80% of South Africans were eligible for the housing subsidy as they earned R3500 or less a month. By 2000, this had grown to 85.4% of the population (Department of Housing, 2003, 9); this increase reflects the rising problems of poverty and unemployment. Irrespective of state programmes for shelter improvement, it should be remembered that perhaps the most significant obstacle to addressing inadequate housing remains South Africa's extreme inequalities of income and wealth. 90% of the population can only afford housing costing less than R190,000 (approximately $27,000) (Rust, 2006, 14). In this context, the majority of South African citizens are dependent on access to the subsidy programme to improve their housing.

The government sought changes in response to these findings. In 2004, the Minister announced a revised National Housing Strategy ‘Breaking New Ground’ which sought to address the housing backlog through a number of measures including the upgrading of informal settlements (Department of Housing, 2004). The withdrawal of large construction groups due to low profit margins (ibid, 5) encouraged new initiatives. From about 2003, state policy had shifted away from private developers to municipalities as the main producers of housing, and insufficient capacity at municipal level was becoming a more significant issue. The 2004 Strategy recognised the need for greater integration within the housing market to make it easier for households to trade up or down. Reflecting some of the original intentions and acknowledging the failure to successfully link subsidy recipients to the formal financial sector, further measures were introduced to assist those earning between R3500 and R7000 ($500 and $1000) to enable access to mortgage finance and the purchase of a unit through the formal market (Porteous, 2005, 35). Links were made with poverty reduction and the programme was represented as one that transferred assets with the anticipation was that new home owners would have additional development options (Lemanski, 2011, 60). Other measures included an increased role for the municipality in improving the provision of services, and the introduction of “a new informal upgrading instrument to support the focused eradication of informal settlements.” (Department of Housing, 2004, 12). In part the interest in informal settlement upgrading emerged because of the need to increase residential densities, improve existing locations and challenge apartheid spatial forms. Huchzermeyer argues that the government's discourse increasingly moved away from house-building towards informal settlement eradication (2011, 115). Although the new policy and programme was announced in 2004, little progress towards the upgrading of informal settlements followed in part due to the lack of fit of this policy within traditional approaches to urban planning (Huchzermeyer, 2006, 51; Huchzermayer 2009, 99).

#### 3.2.1 The People's Housing Process

To support community driven, self-build activities, a People's Housing Process policy was launched by the government in 1998 (Landman & Napier, 2010). The policy is designed to offer greater scope for communities to make decisions for themselves, it also allows them to provide voluntary labour (thereby also improving their skills) and to manage project activities thereby avoiding payments to contractors (Mthembi-Mahanyele, 2001, 4). The People's Housing Process option emerged in part because the local communities linked to the South African Homeless People's Federation demanded a more community-driven collectivised process (Baumann, 2003; Khan & Pieterse, 2006). However, there are concerns that the People's Housing Process has been marginalised with relatively few subsidies being allocated to this option (Miraftab, 2003; Baumann, 2003, 9). Baumann (2003, 10) also highlights problems of formalisation and bureaucracy. These concerns are replicated in critical analysis of the more broadly based housing programme.

### 3.3 Critical perspectives on the South African government housing programme

Despite the scale and benefits of the subsidy programme, concerns have been expressed with respect to location, construction quality, user participation and user involvement. After summarising critiques in the professional literature, the discussion below explores the responses of those in continuing housing need to gain a greater understanding of the difficulties faced by the government in programme implementation.

#### 3.3.1 Subsidy location

A major emphasis in the academic discourse has been on the fragmentation of urban space with many subsidy-financed projects taking place in peri-urban areas. To reduce the need for high expenditure on land, local authorities and developers tend to locate new housing subsidy developments on peripheral land far from economic opportunities, reinforcing the spatial and racial distortions of apartheid and entrenching poverty (Oldfield, 2004; Pieterse, 2006; Zack & Charlton, 2003; and see Schensul & Heller, 2011 for a more general discussion on fragmentation in South Africa). Some of the subsequent problems identified by Zack and Charlton (2003) include lack of access to jobs (ibid, 5), distance from shops, schools, clinics, and recreational amenities (ibid, 30), and the high transport costs associated with distant locations (ibid, 30 and 32). Racial segregation compounds problems of spatial exclusion for low-income households (Pauw & Mncube, 2007).

As a result, some families have moved away from these areas and created a new group of homeless people with no remaining subsidy entitlement (Tomlinson, 2003, 84; Zack & Charlton, 2003). The lack of employment opportunities may encourage households to leave even from better located sites. Lemanski (2011, 66) in a study of a well-located primarily subsidy-financed development in Cape Town finds that almost a quarter of residents have purchased their property (i.e. were not the original beneficiaries). Due to such concerns, the national government now requires that these developments are linked to the statutory municipal Integrated Development Plans (IDP) introduced to assist local authorities to carry out their development role (Harrison, 2008). Plans are developed through a process which allows for the participation of local level stakeholders. All housing developments, whether subsidised or commercial, must now align with the local IDP. However, there are concerns about the effectiveness of these processes in respect of securing urban development including the quality of citizen participation (Todes, Karam, Klug, & Malaza, 2010).

#### 3.3.2 Subsidy size and quality

An on-going issue with subsidy-financed houses has been the size of the unit (Miraftab, 2003). In the late 1990s, minimum house sizes were introduced to address the problems associated with very small dwellings and the present minimum is 40 m^2^. Despite this, it is common to see shacks attached to subsidy houses as households build additional rooms to accommodate their families in part because their shacks may have been larger prior to their move (Lemanski, 2008). There have also been concerns about the quality of housing with inadequate foundations and problems of flooding, cracking walls and other concerns. As a result of these issues, the government introduced a building warranty. Over the period of the subsidy programme there has been an increase in standards with rising specifications.

#### 3.3.3 Participation and beneficiary involvement

The quality of participation in subsidy housing projects has been subject to continuing debate. The initial design of project-linked subsidies assumed that communities would actively participate. However, developers sought exemption (to reduce the time taken in housing delivery), requesting the ability to fast track construction (Miraftab, 2003). Miraftab (2003, 226–227) argues that participation in housing development is limited and residents are unable to insist of their rights to be included in decision-making. This argument is broadly supported by others including Lizarralde and Massyn (2008), Lemanski (2008), Oldfield (2008) and Pieterse (2006).

Swilling (2008) discusses the ways in which social movement organisations have sought to be involved in decision-making and to engage with participatory processes despite these difficulties. He suggests that groups such as the Federation of the Urban Poor (FedUP) have been successful because they have been able to maintain an autonomous organising capacity while engaging with the politics of the city to their own advantage (Swilling, 2008, 508). Miraftab (2003) is broadly in agreement with Swilling and concurs that there are some positive experiences and that social movements are challenging the constraints that residents have faced in participating in housing developments.

#### 3.3.4 Lack of emergency housing

While Huchzermeyer (2004, 2011) has suggested that the rights-based framework is appropriate for analysing the lack of recognition given to informal settlements and related development problems in general the issue of rights has not figured that prominently in academic and professional discussions related to housing need. In part this appears to be because of the scale of the capital subsidy programme for housing. One exception has been the discussion about housing entitlement raised by a court case in 1999 in which Mrs. Grootboom and almost one thousand other adults decided to move from a water-logged area in which they were living onto a vacant hill side set aside for low-income housing (Sachs, 2005). Following eviction by the local authority, the group pursued the council in court arguing that it should meet its constitutional obligations and provide temporary accommodation. After several challenges, the Constitutional Council argued that the housing programme was broadly appropriate to the rights as established in the constitution but there was a need to augment the existing subsidy programme with an emergency housing programme. In 2004, the government introduced measures for those requiring emergency housing including grants for municipalities seeking to provide emergency shelter to residents.

#### 3.3.5 The growth of informality for those still waiting

In addition to concerns about the nature and impact of the capital subsidy programme, there is also a discussion related to the relative lack of scale and continuing housing need. Despite the subsidy-related housing investment taking place in South Africa, the 2001 census identified that 16.4% of households are living in inadequate dwellings and concluded that the absolute scale of need increased between 1996 and 2001 (Statistics South Africa, 2001, 78). The Department of Housing estimated the housing backlog in 2001 to be at 2,784,193 (Department of Housing quoted in Miraftab, 2003, 231). By 2007, it had grown to an estimated 3–4 million houses due to population increase, migration to urban areas, and new household formation (Baumann, 2007). In the absence of alternatives, informal housing continues to be a widely used solution. A report from the South African Institute of Race Relations suggests that informal dwellings are of growing significance and are increasingly being built as backyard shacks in formal areas rather than in informal settlements (SAIRR, 2009).

Between 1996–2007, the total number of households residing in informal dwellings grew by 24.4% from 1.45 million to 1.80 million. During that period, the number of households living in backyard informal dwellings rose by 46% from 403,000 to 590,000. The number of households staying in free-standing informal settlements grew 16% in comparison, from just over 1 million to 1.2 million.^8^

Over this period, backyard informal structures in formal settlements as a proportion of total informal dwellings grew by 18% while those built in informal settlements declined by 7%. There has been a shift away from households living in informal settlements towards the renting of shacks within formal areas, including settlements constructed with subsidy-finance. Lemanski (2009) discusses the problems faced by both groups of households, as well as the benefits that potentially accrue to both parties; she also highlights the lack of a policy response.

At the same time, the number of informal settlements remains substantive. Within these areas, people go about meeting their own housing needs. Without access to the subsidy and unable to afford formal options, families do what is possible to improve their dwellings (Landman & Napier, 2010). In 2005, the Community Organisation Urban Resource Centre (CORC) profiled informal settlements in Johannesburg and identified 131 informal settlements with a population of 692,858 citizens (CORC, 2005). This profiling drew on an earlier study of 102 settlements by the Centre for Applied Legal Studies. The study is believed to include 97% of all informal settlements in the Johannesburg Metropole (ibid, 14). Huchzermeyer (2011, 130–131) further discusses these numbers and highlights the contestation over the scale of informal settlements, and their suitability for upgrading. CORC's exercise was repeated in Cape Town where official maps identified 176 informal settlements within the Metropolitan Area. CORC identified over 200 informal settlements and profiled 183. Twenty one of the settlements identified by the City in the official Metro maps currently being used no longer existed as their residents had been relocated and/or evicted; at the same time, 45 of the informal settlements identified by CORC did not have any recorded identity within the City administration (CORC, 2006, 8). Such findings document the ambivalence of the state with regard to informality.

#### 3.3.6 Collective resistance – evictions and land invasions

Despite the performance to date, citizens exhibit a confidence and trust in respect of government delivery mechanisms with little understanding of the difficulties the government is facing (Smit, 2007; Cherry et al., 2000). On the part of the state, there continues to be a confidence in the capacity to deliver and address basic needs with little considered analysis of the systemic weakness of government programmes.

There is relatively little documentation and academic writing on the tenure struggles of residents in informal settlements. One exception to a general lack of documentation is the work of the South African Homeless People's Federation and the subsequent Federation of the Urban and Rural Poor who have attempted to engage with and hence transform state housing programmes (Baumann, Bolnick, & Mitlin, 2004; Bolnick, 1993, 1996; Khan & Pieterse, 2006; Millstein, Oldfield, & Stokke, 2003; Robins, 2008; Swilling, 2008). In this case, the social movement organisation works closely with a support NGO that helps to facilitate the dissemination of its approach and its work. More recently a body of literature on Abahlali baseMjondolo has emerged (Bryant, 2008; Patel, 2008; Pithouse, 2006, 2008b). A further contribution is the collection of South African movement experiences published in 2006 (Ballard, Habib, & Valodia, 2006) which includes three case studies broadly located in the area of land and housing (Oldfield & Stokke, 2006; Greenberg, 2006; Khan & Pieterse, 2006). A central theme in this literature is the success of these struggles in respect of securing resources from the state. However, Khan and Pieterse (2006, 158–159) highlight the challenge faced by organisations struggling for policy reform within the context of generous state provision (albeit limited in scale); they describe how the leadership of the South African Homeless People's Federation was sceptical that the state would deliver development to the urban poor but, at the same time, they believed that “pragmatic rather than confrontational engagement would yield more fruitful outcomes for the urban poor.” (ibid, 159).

It is difficult to gauge the scale of land invasions that have taken place since 1994. There is some evidence of strategic invasions around election time (see, for example, Skuse & Cousins, 2007). The scale of invasion is indicated by reports of groups affiliated to the South African Homeless People's Federation invading land (despite the preference of the federation to negotiate rather than confront) and examples includes Agrinette Hills (Baumann et al., 2004, 205), Joe Slovo (SDI, 2007) and Ruo Emoh (People's Dialogue on Land & Shelter, 1999). Smit (2007) draws together the conclusions of four regional community workshops on land for the urban poor^9^ and broadly supports the view that land invasion is a significant tactic used by community organisations to secure land. There is also limited information on evictions that are taking place in response to land invasions and informal settlement. Mayekiso (2003, 73) reports that the Gauteng Ministry of Housing evicted 2262 residents. Centre for Housing Rights and Evictions (2006, 31–34) gives details related to evictions from inner-city tenements and informal settlements. But the report emphasises that the scale remains unknown (du Plessis, 2005, 126). Pithouse (2008a) discusses the practices of eviction in Durban and suggests that they contravene the legislation and are illegal; while precise figures are not provided, his discussion suggests that the numbers are significant. The informal settlement profiling exercise in Cape Town found that about 12% (21 of the 176) of informal settlements on official maps no longer existed due to their removal by the authorities (CORC 2006, 8), and the experiences of residents in one informal community is discussed in Thorn and Oldfield (2011).

### 3.4 Conclusion: the challenge for housing policy

While a national housing programme has provided houses for millions of South Africans, a number of criticisms have been made in respect of this programme. For those who have secured a subsidy-financed house, there are notable shortcomings. One of the first problems to be identified was the small size and poor quality of the construction; changes in regulations have now sought to raise standards. Many dwellings have been built in adverse locations with continuing spatial disadvantage. Such locations may reflect the fact that, regardless of the policy, participation in housing programmes is generally superficial due in part to the lack of interest by providers, and the lack of organisation among many households. Primarily as a consequence of such poor locations, there are growing numbers who have left subsidy-finance dwellings.

One of the most serious problems is that, due to the scale of housing need, there are many families that have not been reached. As the state responds to its lack of success in housing provision by restricting the supply of informal solutions through restricting the growth of informal settlements, the continuing need for housing combined with a lack of alternatives means the growth of informal rental solutions in formal housing areas. These households are significantly disadvantaged, not only do they face insecurity and continuing rental payments, they also do not benefit from the subsidies offered by the state to improve access to water and electricity. Despite this, expectations that housing will be provided and a sense of entitlement to receive housing support remain high, both because of the historical significance of housing and evident government intervention in the sector. As a consequence, there has been increasing frustration in South Africa's informal settlements with respect to the lack of effective housing delivery. Families that have not had access to the programme have had to find alternative ways to secure accommodation. Some have turned to participation in social movement organisations to find a way to both address their immediate needs and challenge the scale of collective deprivation.

## 4 Shelter, social movements and housing in South Africa

To inform our understanding of the contribution of South African social movements to addressing shelter poverty (i.e. inadequate access to safe housing with secure tenure and basic services), we considered the literature on social movements, their interaction with the state and their political relations more generally. Research on social movements has been focussed primarily on Europe and the US, and hence our consideration of this literature has necessarily to reflect on the ability of these ideas and related concepts to translate to the global South. There is an emerging literature on social movement activism in the global South and particular in South Africa. In this context, both Ballard et al. (2006) and Thompson and Tapscott (2011) recognise the increasing numbers of South African movement organisations concerned with access to urban basic services and hence their findings are consistent with the conclusion of the first phase of this research project, i.e. that this is an important domain of action for social movements.

### 4.1 Understanding social movements

Historical experience provides evidence of both the successes and failures of social movements (Tilly, 2004). On the one hand, there have been significant advances exemplified by the spread of democratic government and an increasing recognition of the rights of citizens in terms of political freedoms and expanded provision of essential goods and services. On the other hand, specific experiences point to the challenges which movements and/or other groups of organised citizens experience when seeking to advance their cause. The progress of social movements has been theorised through the extensive contributions of Tilly and Tarrow (see for example Tilly, 2004 and Tarrow, 1998), as well as by others who have taken up these and overlapping issues (as summarised in Crossley, 2002). This work helps us to understand the ways in which organised citizens have engaged with political processes and secured progressive change. In terms of social movements, primary focus has been on movements involved in contentious politics, i.e. public articulations of dissent. One notable theoretical approach associated with this work has been the political opportunity structure or process, i.e. the ways in which political opportunities arise and can be taken advantage of by organised citizens. This conceptual framework is widely acknowledged as a starting point for those studying social movements.

While the major focus of Tilly and Tarrow's work and those of succeeding researchers has been on the United States and Europe, there have been some attempts to take these ideas and apply them to the global South. In South Africa, this literature have been taken up by Ballard et al. (2006) and more recently by Thompson and Tapscott (2011) and Mohanty et al. (2011). These ideas have also been explored within a more general deepening of political analysis in the global South. To further such aims Heller and Evans (2010) look at the relevance of Tilly's work on inequality with a three country review that includes South Africa. They acknowledge the importance of such movements in challenging structural disadvantage and securing citizenship and social transformation (ibid, 437), and emphasise the importance of Tilly's “…insistence on contentious politics … as an engine of social change.” (ibid, 438).

In terms of movement goals, across the global South three particularly significant strategies have been used by social movements to shift the state into a more favourable position: new governance systems that open up government processes; new ways of thinking about development which influence public debate as well as programme and policy design; and new ways of engaging the state in programmes designed by social movements (Bebbington & Mitlin, 2006). All involve both governance and resource allocation issues, and the governance implications are particularly strong in the first and last cases which are essentially strategies that aim to realign political relations between institutions. Such impacts go beyond simple effects on “policy”, and these strategies are seeking to influence the nature of state and of the relationships between it and civil society (see Salamon & Anheier, 1998). While Section 3 emphasises the importance of the particular policy context in respect of housing needs, these analyses suggest that our understanding of political progress for social movements working in the area of shelter needs to go beyond this specificity to look at some of the underlying interactions between the movement participants and the state.

### 4.2 Political opportunity structure (process)

One of the most widely used theories to understand the success or otherwise of movements is that of the political opportunities structure (or process). A movement, in this body of literature, has been described as enacting a ‘synthesis of three elements’: sustained public effort (campaign), combinations of forms of public political action (demonstrations, petitions, meetings, media engagement), and public representations of the participants’ worthiness, unity, numbers and commitment to their cause (Tilly, 2004, 3–4). Movements necessarily engage governments to advance their cause and Tarrow (1998) suggests that movement success depends on the underlying conditions for mobilisation and particularly “…the opportunity-threat to challenges and facilitation-repression by authorities.” (ibid, 18). Such political opportunities are elaborated to be “consistent – but necessarily formal, permanent, or national – dimensions of the political struggle that encourage people to engage in contentious politics” (ibid, 19–20). Tarrow (1998, 3) recognises that movements use collective action to open up the possibility of further actions and advance political opportunities. But he also recognises that such opportunities need to be understood as being much wider than simply those arising from the consequences of mobilisation; for example, progress is influenced by the nature and depth of cultural practices, and by the breadth and depth of horizontal networks and alliances with more powerful agencies.

In this literature, political opportunities are represented as dynamic and interactive as actions and responses open up and close down different potentials. Tarrow (1998, 19) describes how influence is achieved when he argues that “people engage in contentious politics when patterns of opportunities and change and then, by strategically employing repertoires of collective action, create new opportunities, which are used by others in widening cycles of contention”. However, it is not necessarily the case that opportunities build to a critical mass, and Goodwin, Jasper, and Khattra (1999, 53) stress both the iterative nature of engagements and uncertainty of outcomes when they suggest that “[E]ach side tries to surprise, undermine, and discredit the other.”

The potential opportunities that are referred to are varied and include increased external resources, reduced costs of contention, existing and new alliances, the vulnerability of elites, improved access to institutions, reduced capacity for repression, increasing access, the need for political leaders to have new sources of support, divided elites, influential allies, low state strength, ineffective and illegitimate state repression, and international support (Goldstone, 2004, 347; Goodwin et al., 1999, 32–33). In understanding which opportunities may be important in any given context, it is suggested that those which influence the strength of the state, the prevailing strategies of the state and its level of repressiveness should be given particular significance (Tarrow, 1998, 73). It is also acknowledged that relations with influential allies (groups and individuals) are important in helping movement leaders manage a shift from a consciousness of grievances to a more pro-active agency able to create and use opportunities (see Gamson citied in Tarrow, ibid, 79 and 104). In this discussion and others, there is an acknowledgement of the importance of longer-term processes, rather than specific events, and on the consciousness of the oppressed both with respect to who is to blame, and their understanding of what is a strategic response to a given situation (McAdam quoted in Crossley, 2002, 113–114). Opportunities are not fixed but are created by movements through their ability to understand, act and catalyse a response from the state.

However, there are concerns that such arguments are too general to be meaningful and that opportunities can be whatever is identified in a retrospective analysis as being of significance and hence of little help in understanding causal relations and offering explanations. Goldstone (2004) argues that opportunities need to be more finely defined (ibid, 348), and he suggests that what is critical to movement success is how the demands of protest movements and perceived underlying interests of their members are taken up by politicians (ibid, 354). He elaborates that what is important are the “complex relationships” between groups including state agencies, opposition movements, the leaders and followers and potential supporters who are yet to be organised (ibid, 354). He also suggests that the focus on contention is too restrictive and the analysis of movement activities should focus both on protest and on less contentious tactics to influence government (ibid, 340). Reviewing movement activity in the US from 1800, Goldstone suggests that the political opportunity structure has three main weaknesses: it over-emphasises the contribution of states and underplays that of economy, movement coalitions and other relevant domains of action; it concentrates on opportunity and neglects adversity; and it draws attention to structure and neglects dynamics that are “more fluid and relational.” (ibid, 356). He argues that the approach can be strengthened by giving greater attention to a number of other factors including other movements and counter-movements, political and economic institutions, state authorities and political actors, various elites, various publics, symbolic and value orientations, and critical events (ibid, 357). Further concerns with the approach have been an over-concentration on explicit political engagement rather than a broader set of social relations (Goodwin et al., 1999, 35). Related to this has been recognition that movements may be important not just for their direct impacts on state policy but also because of their challenge to dominant cultural codes and their ability to support alternative collective identities (Koopmans, 1999, 98). Such research highlights both the significance of contentious and non-contentious politics to advancing the interests of movements and their members, and the potential offered by activities aimed at making the broader social context more favourable to the achievement of movement goals and objectives.

One challenge in using these theoretical and conceptual insights to interpret events in this study is that they have been developed primarily for the global North (Tarrow, 1998, 19). However, there have been a number of recent efforts to use these frameworks to understand the progress of social movements in South Africa and they are discussed in the following sub-section.

### 4.3 Political opportunity structure in the global south

Thompson and Tapscott (2011, 5) explore the political opportunity structure approach when studying social movements in Brazil, India and South Africa, and conclude that the approach has relevance to understanding outcomes and activities. They suggest social movements in the South are weaker than in the North because political control is stronger and/or because citizenship is not understood and hence awareness of citizenship does not lead to organisation and mobilisation. They also argue that while the attainment of rights was, in the North, the result of social movement activity, this is not necessarily the case in the South. As a result, in countries such as South Africa, movement members may have their rights acknowledged but still not benefit from substantive material redistribution (ibid, 2). For these and other contextual differences, they argue that conclusions from studies of movements in the global North may not fit well with Southern contexts as they may “…presume levels of political identity and strategic sophistication that are often absent amongst movements comprised of people existing on the margins of survival. For such communities it is often merely a question of political opportunity, driven by desperation that leads to collective action rather than a conscious framing of options.” (ibid, 7). Ballard, Habib, Valodia, and Zuern's (2005) and Ballard et al.’s (2006) discuss social movement theories in the context of South African political activity, and agree on the relevance of the political opportunity structure approach. These authors adopt the broader interpretation of the theory summarised in the sub-section above, and incorporate both an awareness of the importance of networks and available political and material resources, and a recognition of the importance of identity and “cultural frames including shared meanings, symbols and discourses” (2006, 6).

A particular theme in this literature on social movements in South Africa is the purpose of the political opportunities that are sought. Ballard et al. (2006) draw several further conclusions that elaborate the perspectives of social movement activists in South Africa. While recognising ideological distinctions between reformist and revolutionary objectives, they suggest that whatever the preferences of the leadership the members of urban movements, even those of radical movements, may participate in the movement but at the same time support the ANC (i.e. reform rather than revolution). This willingness of citizens to both participate in movement struggles and support the ANC is agreed by Matlala and Bénit-Gbaffou (2012). Present movement struggles are, Ballard et al. argue, very much located in both on-going hardship and government efforts to address poverty and hence are neither visionary nor utopian (ibid, 402). Perhaps as a result of this grounded engagement, Ballard et al. (2006) argue that, whatever the academic discourse, in practice there is a synergy between protest towards and negotiation with the state in South Africa (ibid, 404). They suggest that few movements subscribe only to either adversarial politics or to collaboration with the state, and in practice most follow multiple strategies (ibid, 405–406). Thompson and Tapscott (2011) argue that, notwithstanding the limited depth of conceptual understanding shown by activists, movement organisations are able to challenge state power and influence policy and programmatic outcomes. However, they also suggest that movement leaders may be more concerned to change the position of their members within the political order and hence secure improvements, rather than change the order itself (i.e. these authors agree with the conclusion that the emphasis is on reform rather than revolution). This specific conclusion is notably similar to McFarlane's (2004, 911) following his study of an Indian movement organisation active in shelter issues.

In terms of the specificities of such engagements, Thompson and Tapscott (2011, 16) use a different academic framework to discuss overlapping themes; they suggest that ‘invited spaces’ for state-citizen engagement appear to be less significant than mobilisations outside of these spaces (i.e. by implication that contest is more important that collaboration). Mohanty et al. (2011) and Bénit-Gbaffou and Piper (2012, 177) are broadly pessimistic about the opportunities offered by ‘invited’ spaces in South Africa; these authors suggest that while they improve political inclusion, there is less evidence that they result in resource redistribution. However, a more complex picture emerges from the studies included within the Ballard et al. (2006) edited volume with many organisations moving into and out of such spaces (and some resources gains) as they pursue their needs and interests.

Robins (2008) reinforces this conceptualisation of a set of pragmatic movement organisations strategising to find a way through a range of political opportunities to access state resources. He draws on examples from urban and rural areas to argue that local communities and movement organisations use multiple strategies to influence political leaders including traditional authorities, patrons and religious figures alongside ANC members (ibid, 6). Consistent with the earlier studies of social movements in the global North, he argues that such strategies create political opportunities and further opportunities are also opened through the alliances that social movement organisations have with support professional agencies such as NGOs (ibid, 17). A diversity of strategies emerges because movement organisations have to “engage simultaneously in multiple domains of politics, including those of the state, NGOs, religious organisations and powerful patrons and ‘big men’.” (ibid, 83). Robins (2008) studies the South African Homeless People's Federation in one greenfield development in Cape Town. (FedUP, one of the movement organisations studied in eThekwini emerged following a split within the Federation.) He argues that Federation leaders use ANC networks to secure resources (through patronage networks) and access local authority technical expertise (ibid, 96). Like Ballard et al. (2006), he concludes that movement organisations in South Africa have achieved some success, although he is concerned about their capacity to maintain activism in the longer-term. By implication he also emphasises the importance of dual strategies of contention and collaboration.

In terms of the broad orientation of movements and whether they are concerned with “old” social movement goals of redistribution or ‘new’ social movement goals of recognition, there is a broad agreement that both are relevant (Ballard et al., 2006, 410; Mohanty et al., 2011, 27; Thompson & Tapscott, 2011, 6). Mobilisations have a strong identity focus, and both goals are sought simultaneously (Mohanty et al., 2011, 27).

As noted above, a further contribution to the debate about the relevance of the work of Northern social movement theorists to the global South is Heller and Evans’ (2010) discussion of the significance of Charles Tilly's work for understanding social chance in urban centres in Brazil, India and South Africa. This paper returns to an earlier critique of the political opportunity structure approach and argues for recognition of the structural constraints within which opportunities are created, and specifically the need to understand the city as a political entity, and the significance of relations between cities and the national government for social progress. They acknowledge the relevance of movements such as the Indian Alliance (who share with FedUP an affiliation to the transnational network, Shack/Slum Dwellers International) in being able to change the politics in the city and secure some gains. They view such successes as taking place despite restricted political opportunities in Mumbai with clientelist politics, strong middle-class interests well able to represent themselves and other powerful political groups including organised crime (ibid, 442). In South Africa, they suggest that the context is more favourable than India as urban centres have more autonomy. However, they also suggest that new inequalities in political participation have been constructed since 1994 with increased state managerialism and a weakening of civil society (ibid, 443), and the urban poor have faced further difficulties with growing wage inequalities and continuing unemployment. Despite this, they recognise that community-based social movements are becoming increasingly active and offer positive prospects for greater democratisation and citizenship (ibid, 444). Their analysis highlights the critical relationship between individuals, their group identities and the state; and hence the importance of changing such relationships if more positive political opportunities are to be secured.

Heller and Evans (2010) also highlight the importance of the “urban” location of social movement activism. This theme of urban politics including the relative lack of autonomy of cities to establish their own policy direction and the implications for social movements is previously highlighted by Castells (1983) who agrees that it is a constraint on the scale and substance of the change that can be achieved. More generally, the urban context emerges as significant for a number of authors writing on social movements in the global South; this is a context characterised by the relatively powerlessness of these communities and their dependence on favourable opportunities (Thompson & Tapscott, 2011, 7), the willingness of commercial enterprises to challenge the political status quo in their own interests (Castells, 1983), the consequence of informalisation on labour relations in India (Agarwala, 2006), and the balance of power between city governments and the nation state (Heller & Evans, 2010). Heller and Evans (2010, 443) argue that South African city politics is characterised by a powerful central state that resists participatory processes – and in so doing emphasise the importance of decision-making at the level of local government. As discussed in the preceding section, this theme has also been recognised within the literature on housing policy and local government in South Africa.

Hence, while the political opportunity structure was developed in the global North, it has been used by a number of authors to understand social movements in South Africa with significant support for the approach. More generally, there is a rejection of the division between old and new social movements, and a claim that both issues of identity and distribution are important for movements that are presently active. In terms of the literature on social movements in South Africa, there is a cautious optimism and recognition that some limited gains have been achieved, hence movements have been seen to both create opportunities and use those that exist. In respect of achievements in poverty reduction and inequality, Seekings and Nattrass (2006, 341) in a general commentary these issues in South Africa are more pessimistic than these movement-focussed studies and argue that government policies have exacerbated unemployment and resulted in continuing inequality. These authors argue that they find themselves having to explain to others “…why there was not more political pressure to transform the distributive regime.” (ibid, 341).

### 4.4 Conclusion: themes in the social movement literature

With respect to the specific strategies that movements may use for engagement, there is considerable debate on the relative effectiveness of collaboration (negotiation) and confrontation and this is evident in the discussion above. This theme recurs across the literature: in discussions of civil rights movements and poverty programmes in the US South, indigenous movements in Ecuador and Bolivia (Lucero, 2005), a range of social movements in South Africa (Ballard et al., 2006), urban movements in India (McFarlane, 2004), and rural movements in the Brazilian Amazon (Schmink, 2006). In particular, movements debate and argue about how far their strategies should be conciliatory or conflictive. Both within a given movement over time, and across movements at a point in time, the pattern is that ‘social movements’ engagements with the state fall on a continuum between in-system collaborative interactions on the one extreme and out-of-system adversarial relations on the other” (Ballard et al., 2005: 629). While much emphasis has been placed on contention (Crossley, 2002, 108; McAdam, Sidney, & Charles, 2001), as noted above alternative strategies to secure political advancement have been recognised. While the political opportunities structure approach places emphasis on building a contentious process, when the emphasis on opportunity is replicated by others although the view of what constitutes an opportunity differs.

Relations with political parties also emerge as a critical theme both within discussions about the political opportunity structure and more generally in respect of what helps movements advance their cause. Movements, Goldstone (2004) suggests, have been wrongly viewed as competition and alternative to party politics; while some predict that movements will be institutionalised into the political system, he argues they are symbiotic and will continue to exist side by side (ibid, 336–339). While movement analysts emphasise the importance of alliances with political parties, there are also warnings about the need to maintain autonomy from party politics and avoid institutionalisation (Castells, 1983). The strength of movements is considered to be related to their ability to manage these relations, taking into consideration the ways in which the parties respond to movement-based political opposition and the willingness of citizens to participate in movement activities (Thompson & Tapscott, 2011; Heller & Evans, 2010).

As noted above, the specificities of urban politics including both the nature of political relations within cities, and the dependence of city governments on national political processes is recognised. A further part of the urban context is that of culture, and the role of culture in social movements is acknowledged by scholars who work in both the global North and South. McAdam et al. (2001) explicitly goes beyond the frame of reference of North America and Europe when they emphasise the importance of culture in explaining forms of mobilisation (and related processes) (ibid, 23–24); in their analysis, these authors recognise that culture helps to define both what is contentious and the degree to which it is contentious politics is considered to be legitimate practice. As noted above, understanding the experiences of housing provision in South Africa requires an understanding of culture and particularly the influence of history on the understanding of housing need and the practice of housing struggle.

The following section briefly introduces the shelter situation in eThekwini, the agencies involved in addressing shelter needs in the city, and analyses the housing problem there as understood by these agencies. Section 6 then reports on the strategies used by the movement organisations as they seek to realise the needs and interests of their members.

## 5 Shelter in eThekwini/Durban: history, agencies and issues

This section introduces housing provision in the city, briefly describes the civil society agencies included in the research and summarises the key shelter problems as elaborated by the interviewees.

### 5.1 A brief historical introduction

Durban's spatial development has been marked by apartheid and as early as 1880s planners began to create particular zones for different racial groups (Pithouse, 2008a, 20, and see Schensul & Heller, 2011). In the following decades, the port city grew to be the second largest urban centre in the country. Housing problems were evident as low wages combined with adverse policies that restricted livelihood opportunities for African families (Pithouse, 2008a; Marx & Charlton, 2003). Segregation intensified from the 1940s with black South Africans being pushed away from the better locations (Marx & Charlton, 2003; Schensul & Heller, 2011). Changes in regulations and planning legislation were used both to include and exclude particular residents and favour industrial interests (Scott, 2003, 245). However, the labour requirements of commercial enterprises helped to ensure that some informal settlements remained and even in the late 1940s there were still 70,000 people living in shacks within the city (Pithouse, 2008a, 32). In the 1950s, the Group Areas Act provided the basis for forced mass removals of Indians and Africans and throughout the late 1950s and 1960s, the clearances and evictions continued (ibid, 34).

By 1984, there were an estimated 1 million living in shacks in and around the city; this increased to 1.7 million in 1988 following the official abandonment of influx control (Pithouse, 2008a, 38–39). Informal settlement formation continued with difficulty and new informal settlements were small and/or on land at risk of floods or landslides. With democratisation in 1994, funds from the new housing policy were made available for shelter improvements. However, progress appears to have been slow. In terms of the population mix, Schensul and Heller (2011) argue that there was some desegregation of neighbourhoods between 1996 and 2001, although in two-thirds of neighbourhoods there was very little change. They conclude that in 2001 70% of Africans remained in poorly serviced and adversely located areas with few employment opportunities. By 2002 the housing backlog was estimated to be 305,000 units with 108 informal settlements (Marx & Charlton, 2003, 1 and 28), and only one-third of shacks were considered to be on relatively well-located and safe land. Estimates for 2007 suggest that 4.5% (37,500) of the 833,859 households within eThekwini Metropolitan Area lived in informal dwellings in backyards (formal settlements) with a further 4.5% living in a room within a shared formal property, 67.7% lived as individual households in formal settlements, 12.6% were in informal dwellings in informal settlements, with the remainder being in traditional dwellings (6.4%) and temporary accommodation such as caravans, tents and hostels (4.2%) (SAIRR, 2009). Assuming that those in need include informal dwellings in both backyards and informal settlements, and those in shared and temporary accommodation (but not including those in traditional dwellings), the housing backlog was just under 225,000 households. In 2007, there were an estimated 17.1% of households in informal dwellings with 12.8% of residents lacking electricity connections. Numbers without piped water and without access to improved sanitation are very small (2.5 and 3.3% respectively) (SAIRR, 2009), although some of these are shared facilities. In June 2010, the city council estimated the housing backlog was 368,000 dwellings with 239,000 households in informal settlements and 45,000 living in backyard shacks (up from 37,500 in 2007).^10^

The eThekwini Municipality was created at the end of 2000 with the urban boundaries being redrawn to recognise linkages and inter-dependencies between different districts and “…the need to redistribute resources from a relatively wealthy centre to a much poorer periphery.” (Marx & Charlton, 2003, 3). Both before and after this there were tensions between national, provincial and metropolitan authorities in the delivery of improved housing. The city government sought to secure housing subsidy finance from the province to address their residents’ needs with the accreditation they needed to undertake subsidy-financed housing projects themselves (Charlton, 2003, 268 and 272–273). The Metro Housing Service Unit has been established in 1997 and in 2001 it was merged into the new uni-city (eThekwini Municipality). Charlton describes a number of tensions prevailing during this period most notably the allocation of provincial subsidies on a project basis (rather than a regular programmatic commitment), the lack of local influence over national policy, policy differences between the national and provincial level, and conflicts between housing and other policies (such as environmental planning requirements) (ibid, 268). Metro Housing staff coordinated different agencies to ensure an integrated programme and put in place additional funding in place to top up the subsidies, but faced difficulties including the high cost of construction due to the topography, and lack of available and well-located land. In terms of the more general concerns about the housing policy discussed in Section 3, staff recognised the need for informal settlement upgrading (Charlton, 2006, 52) and from 2000 were aware that some beneficiaries of the housing subsidy-financed units were leaving peripherally located Greenfield projects to return to informal settlements (Charlton, 2003, 273). Since 2002 it has not been possible for commercial contractors to be the developers and hence the municipality has been responsible for providing oversight and overall management, completing housing developments through contracting a range of companies. In 2011, the city council argued that its major housing challenges were to create a more integrated city with greater acceptance of lower-income citizens by the residents of higher-income neighbourhoods and an improved transport network (see note 10). They acknowledge continuing growth in the housing backlog, despite housing completions. Low-income and lower-middle income residential areas need investments in education, employment opportunities and improved public transport provision. Their conclusions are reinforced by Schensul and Heller (2011, 105) who conclude that while some subsidy investments have been effective in creating new opportunities for those without adequate housing, on balance “existing housing markets are deeply stratified across the city and set very tight parameters on who can move.”

In terms of policy issues specifically related to informal settlements, the Centre for Housing Rights and Evictions (2006) reports that, in 2001, the KwaZulu-Natal provincial housing minister Dumisani Makhaye began to talk of “slum clearance” and introduced a policy to achieve this goal. A particularly contentious issue has been the Elimination and Prevention of the Re-emergence of Slums Act passed by the provincial authorities in 2007. This Act seeks to “to provide for measures for the prevention of the re-emergence of slums, to provide for the upgrading and control of slums; and to provide for matters connected therewith.” (Sabinet Online, Extraordinary Provincial Gazette of KwaZulu-Natal, 2 August 2007). Tolsi (2009), writing in the Mail and Guardian Online, argues that the Act “allows for municipalities to fine or jail private landowners for not evicting unlawful inhabitants from their land in a time frame determined by the provincial housing minister.” The Act focuses on the illegal and unlawful occupation of land and buildings (clause 2.1) and explicitly includes those renting premises that have not been approved by the municipality under the National Building Regulations and Building Standards Act 1977 (clause 5). Hence if households rent sub-standard accommodation (perhaps because they cannot afford any other), the Act requires them to be evicted. The Act also requires municipalities to identify “any land or building … likely to become a slum” and require their upgrading; if they are not upgraded, then the owner or person in charge commits an offence (clause 14). All owners and persons in charge of vacant land or building must “take reasonable steps to prevent the unlawful occupation” or they also commit an offence. If the land or building is already occupied by unlawful occupiers, they must be evicted (clause 16). The penalties associated with such offences are “a fine not exceeding R20,000 or imprisonment for a period not exceeding 5 years” (clause 21). Even without the Act, there was agreement among interviewees that eThekwini authorities can control the expansion of shack settlements. Officials explained that the municipality employs informal settlement monitors who inform on new land invasions, and the construction and extension of shacks. This does not extend to backyard shacks in formal areas which are considered to be the responsibility of the owner.

According to both Marie Huchzermeyer (Associate Professor at Wits University) and Bonile Ngqiyaza (The Star, 2009), the Elimination and Prevention of the Re-emergence of Slums Act has not been implemented although it was placed on the provincial statute books on 18th July 2007. AbM challenged the Act during its formulation and then once it became legislation. SDI, the international network to which FedUP is affiliated, has also come out against the Act (Weekend Witness, 2009).^11^ AbM's efforts took the case all the way to the Constitutional Court and a judgement was issued in October 2009^12^ declaring parts of the Act to be inconsistent with the Constitution and therefore invalid.^13^ An undated media statement of the Department of Housing's web site (http://www.housing.gov.za/, accessed Monday, 16 February 2009) illustrates the state's earlier reaction to AbM's attempt to have the Act declared unconstitutional, and highlights differences in perspective. The Department quoted reported that “Judge President, Tshabalala JP concluded that the Province of KwaZulu-Natal must be applauded for attempting to deal with the problem of slums conditions. ‘The Slums Act makes things more orderly in this province and the Act must be given a chance to show off its potential to help deal with problems of slums and slum conditions’.”

### 5.2 Introduction to the civil society agencies

Using the snowballing method described in Section 2, we identified a number of social movement organisations and their support organisations. They are briefly introduced here.

The Federation of the Urban and Rural Poor is a social movement organisations that works in alliance with CORC (Community Organisation Resource Centre) and uTshani Fund to network low-income community organisations and support them to address their shelter needs. FedUP mobilises the community through saving schemes and supports their local activities. Saving schemes elect their own leadership primarily drawing on their mainly female membership. One interviewee explained that FedUP is not only concerned about access to land and housing, but is using these objectives as an entry point to tackle poverty. FedUP puts its membership tens of thousands and talked about having 80,000 members at its peak with some decline since then. FedUP is member of the transnational network, Shack/Slum Dwellers International (SDI), and is committed to the principles of this collective which include maximising women's participation. The uTshani Fund was established in 1995 as a Section 21 company to enable the South African Homeless People's Federation to secure loan capital for subsidy-financed housing construction; following the split in this movement organisation, uTshani has continued working with FedUP. The Fund has increasingly become a conduit for state subsidies (rather than a pre-finance facility) and has developed its technical capacity to meet the increasingly stringent controls related to subsidy-financed construction. The uDondolo Trust has recently been established to administer donor funds for FedUP's community to community exchanges and other expenses such as local centre administration. CORC provides support to communities seeking to innovate and develop their own solutions to their problems. It supports numerous FedUP activities particularly enumerations and city-wide surveys, and also supports other communities. In eThekwini, FedUP sometimes augments professional support from COURC and uTshani with expertise from other NGOs.

Abahlali baseMjondolo^14^ is a network representing 34 settlements in Durban.^15^ The organisation began in Kennedy Road, and its inception was influenced by the Kennedy Road Development Committee. Interviewees traced the formation of this organisation back to 1985. The network was formally launched as Abahlali baseMjondolo (AbM) in 2005. Initially AbM was concerned with access to land and housing and preventing evictions; it continues to have a focus on shelter but now frames these issues within the concepts of dignity and recognition. AbM works with the Western Cape Anti-Eviction Campaign (Cape Town), the Landless People's Movement (Johannesburg) and the Rural Network (KZN), all of which are networked together as the Poor People's Alliance. AbM has worked with professional organisations in specific projects or programmes of work; for example, with Centre for Civil Society (University of KwaZulu-Natal) and with Open Democracy to produce a film. More recently, leaders have developed a close working relationship with the Church Land Programme, a NGO that seeks to learn from them and support their activities. Members also work with the Centre for Applied Legal Studies and the Legal Resources Centre.

The Church Land Programme grew out of NGOs working in rural development to identify land owned by churches and secure land for the landless. It is an independent organisation constituted by the Methodist, Anglican and Catholic churches, and ecumenical groups. The Programme currently has an alliance with the Landless People's Movement (Gauteng), the Anti-Eviction Campaign (Western Cape) and the Rural Network, and works with the Poor People's Alliance. In its work, the organisation is now also engaging with the Department of Land Affairs. It provides significant support to AbM including a contribution towards the costs of AbM members who are studying at the Centre for Adult Education (University of KwaZulu-Natal).

The Build Environment Support Group (BESG) is an NGO established in 1983 by staff from the Architecture Department at the University of KwaZulu-Natal to provide technical support to low-income groups in need of housing. Such housing project implementation now only constitutes 3% of its work and there is a much greater current focus on livelihood issues such as health and agriculture. Staff seek to support organisations of low-income and disadvantaged households, and work in alliance with organisations like Children in Distress Network looking at children's rights.

The Project Preparation Trust (PPT) is an NGO formed in 1993 to prepare low-income communities for housing provision. Its mission has since enlarged to include programmes in local economic development, special needs housing (orphanages) and food security. The Trust operates largely in KwaZulu-Natal and focuses on providing services for clients. Recently staff have worked with AbM to address problems faced by shack dwellers in 14 AbM settlements.

### 5.3 Understanding the housing challenge in eThekwini

There is a notable consistency in the analysis of housing problems in the city across many interviewees. The views and perceptions reported here were gathered when interviewees talked about their understanding of shelter need. Three themes emerged, the first of which is the regulated and professionalised nature of subsidy-financed residential developments and associated consequences for citizen and state relations. The second theme is the lack of well-located residential land, and the related pressure for relocation and/or densification within some informal settlements that are being upgraded, and the use of transit housing and alternative sites. The third theme is the scale of housing programmes relative to housing need in the city. While there is a broad consensus on the part of both state and social movement associated interviewees about the nature of the challenge, there is less agreement on preferred solutions.

The housing sector in eThekwini is considered to be highly regulated and professionalised. The perspective of senior staff in both provincial and city government is that there are numerous policies and “lots of rules” which control the nature and outcome of subsidy-financed housing projects. The head of housing within the municipality exemplified this issue when he discussed the problems faced by one local FedUP group wishing to construct double storey houses (a shift encouraged by the municipality because of the need for densification) but who faced problems with construction regulations that prevented the use of cost-effective suspended wooden floors which he explained are “used everywhere but not allowed here.” A senior provincial member of staff suggested with concern that the level of regulation may deter civil society groups from being more involved in direct provision particularly in relation to the People's Housing Process (PHP) and its successor, the ePHP. One NGO staff member who works with FedUP highlighted how the increasing weight of regulations causes frustration for community groups who faced consistent delays because local authority state “tell us to wait for inspections…” There is some suspicion among Federation groups that they are subject to more stringent inspections than municipally managed housing developments. Some comments were more favourably inclined towards this approach: the informal settlements officer in the municipal planning department concurs with the need to support an effective ePHP but argues in favour of the regulatory framework elaborating that it is “local government's mandate to house the people” – reflecting the underlying tension between a providing and an empowering state.^16^

A second theme identified is the lack of well-located land for low-income housing and associated difficulties. There are an estimated 514 informal settlements in the city.^17^ As noted above, settlements in eThekwini, particularly those closer to the city centre, are high density and it is often not possible to accommodate all residents and comply with the minimum criteria stipulated by the city in the case of single-storey detached dwellings when redevelopment takes place.^18^ As Huchzermeyer (2011) elaborates, in South Africa such upgrading first involves creation of greenfield sites on the land previously occupied, with subsequent rebuilding of houses. The problems are exacerbated because one shack may contain several families with adult sons and daughters continuing to live with their parents even when they have a family of their own. To illustrate the problem, the Community Liaison Officer at Cato Crest (one well-located informal settlement currently awaiting redevelopment) explained that of the 7500 households being supported with improved accommodation, only 1500 households can be returned to Cato Crest itself where work is within walking distance. The rebuilding of existing settlements and the need for relocation requires the use of transit housing. In some cases the stay is only for several months while new houses are built on existing locations but in other cases transit housing is used for households being relocated to other sites and delays are considerable. Even a temporary move into transit accommodation for those who are able to return to Cato Crest can be very problematic because of low-incomes. The Community Liaison Officer elaborated thus: “The monthly wage is generally about R1500 (US$210). Now they are moved 2 km to Ridgeway [the location of the transit accommodation] and have to pay R6 to get back. This is for one trip.” Hence to remain in existing employment during a period of temporary relocation requires an expenditure of R240 a month on transport. Moreover, both municipal officials and civil society representatives are concerned about conditions. Officials commented that “conditions in the shacks are very bad, [the transit housing is] little bit better than what they have” and “16 square meters, corrugated iron, community facilities, very high-density, confined area. It can be very hot. Not ideal.”

In terms of permanent relocation, council officials suggested that some relocation sites may become strategic residential areas due to urban growth and government investment; however, they acknowledge the immediate and longer-term difficulties that result from relocation. While there is no maximum travel time or distance from the original site for those being relocated, the staff member responsible for informal settlements stressed that “relocation is a last resort.” Civil society interviewees were critical about the consequences of relocation for local citizens as they find it difficult to obtain work and access to services is often poor. A particular problem is that the local authority is not able to provide transport or schools as these services are managed at higher levels of government (although they are able to provide health clinics). There are frequently delays in these investments and children may have to travel considerable distances or stay with relatives. Informal transport costs, if available, are likely to be high. FedUP members recognise these problems and respond by saying that they are careful to explain the difficulties to families interested in relocation as and when opportunities emerge. They consider that it should be up to their members whether or not they fight to remain in existing settlements or relocate. AbM members also recognised that some relocation may be needed in the case of some settlements, and argued that proximate land to existing settlements is needed.

An alternative to relocation is densification including the construction of medium-rise apartments. There are mixed views on the desirability of this option. Council officials recognise the need but are concerned about the feasibility. One municipal official suggested that “collective ownership does not work even for higher-income households. There is a need to provide everyone with their own plot.” FedUP have sought to experiment with double-storey houses in high-density settlements and have been able to identify members that are willing to consider these options; however progress in these innovations has been slow.

The third theme consistently raised by interviewees is the inadequate scale of the housing programme.^19^ In recent years, the municipality has completed an annual average of between 16,000 and 18,000 subsidy-financed dwellings. However, municipal staff told us that the water services department estimate that 20,000 households are coming into the city each year, a figure they consider to be reasonably accurate. The policy of the Council is to be “slum free” by 2014. Staff explained that if all rural and backyard shacks are to be upgraded and there is an annual 2% increase in “slum” populations then, if the municipality maintains its current building programme of 16,000 units a year, eThekwini will be slum free in 2022. The building programme needs to increase to about 33,000 a year for the city to be slum free in 2016. Neither of these predictions take into account the estimated 20,000 households moving to the city each year – the 2% increase is equivalent to 4–5000 households a year. This is the context in which the controversial Elimination and Prevention of the Re-emergence of Slums Act (2007) was attempted (see below).

Municipal staff also recognise the problem of residents who have no subsidy entitlement. We had discussions with City officials about what happens to such households in informal settlements that are being upgraded. It was explained to us that they would be allowed to remain in the settlement although they would not have a house constructed for them. However, other council staff and community members did not agree that this is the practice and said that they were not allowed to return.

In the absence of substantive improvements in informal settlements, the city has introduced a programme for standpipes and toilet blocks to provide some relief for the residents of long-established and inadequately serviced informal settlements. The municipal decided in 2001 to stop upgrading electricity services in informal settlements prior to comprehensive upgrading.^20^

What is notable about the views of interviewees is the concentration of comments on a relatively small number of themes and the high degree of the consensus about the nature of problems that the city faces. There is a concurrence of opinion that the scale of regulation and formalisation is dysfunctional, that problems associated with relocation are significant and need to be addressed with the incidence of relocation being minimised, and that the scale of housing provision should be increased. In terms of differences in opinion, in respect of the first of these themes, state officials are keener on controlling the process on housing development (albeit with more skill than at present) and the civil society organisations challenge the growing formalisation with multiple demands that the process be more respectful of the rights and capacities of local citizens. Moving to the second theme, the differences of opinion include greater emphasis on the possible benefits of relocation (for example, to areas close to the new airport) on the part of officials, and greater emphasis on the difficulties and costs faced by families by the civil society interviewees. The former see relocation as a necessary evil, while for the latter it involves unacceptable burdens for families and should be minimised, avoided and/or resisted. Differences of opinion in respect of the inadequate scale of the programme are related to the perceived causes. For civil society organisations, the lack of scale relates to the professionalisation and formalisation of the programme, and the associated delays, together with an inadequate financial commitment and lack of land availability in well-located neighbourhoods. For several state officials, the problem lies with continuing in-migration to the city.

There was one difference in the perceived problem analysis. Civil society interviewees emphasised that housing is only a small part of what is required and that there is also a need for employment, access to services and assistance with food security. The lack of similar arguments on the part of municipal officials may reflect the fact that we only interviewed those with responsibility for housing.

While there are differences in their approach to acquiring land and housing, both AbM and FedUP members emphasise their identity as shack dwellers and their demand for recognition and respect. In this context, improved housing is a contribution to a more comprehensive approach to poverty and inequality. Patrick Magebula, President of FedUP argued with direct reference to the perspectives that lay behind the Elimination and Prevention of the Re-emergence of Slums Act that one of FedUP's objectives is “Shaking off the stigma that we are land grabbers”. For him, securing housing is a struggle for “dignity” and the search for dignity also involves the recognition that low-income people can manage their own subsidy funds and design the houses that they want to live in. He argues that one of FedUP's primary struggles is to convince the state that low-income citizens can build their own lives through managing their own resources. S’bu Zikode (President of AbM) elaborated a similar perspective and also demanded greater respect:

As we began to proceed with our struggle, we realised that many of those in the Council thought that people in shacks could not think for themselves. We face many problems but perhaps the most devastating treatment was that your voice would not be heard. This became a fight for recognition. In a way, we have been diverted from the fight for land and housing into a fight for human dignity.

### 5.4 Conclusion

In respect of housing development, the experiences in eThekwini resonate with those described in Section 3. As noted earlier, there is a diverse and active social movement anxious to improve the quality of shelter. However, the scale of subsidy housing is inadequate. There are particular problems associated with the redevelopment of informal settlements, and related issues of de-densification, relocation and displacement. While the quality concerns related to subsidy-finance construction have been addressed, other problems remain. The interest in an engagement with civil society is recognised particularly at senior levels in government and also acknowledged by more junior staff; but it is, in practice, difficult to realise. A highly technical approach means that communities cannot easily be involved in construction, and in on-going developments it appears that a considerable effort is put into mediating the boundaries between community activities and local authority regulations. At the same time, this difficulty reflects a more deeply rooted contradiction about the roles of civil society and the state. For the social movement organisations themselves, the failure to address these needs reflects prejudice and discrimination towards households with low-incomes. Meeting housing need is therefore an issue of social justice.

There is a broad consensus on the problems facing those in housing need in eThekwini and more general housing challenges in the city. There is also considerable consistency between interviewees from movement organisations and the state in their understanding of the solution, the implementation of the state housing subsidy scheme. While movement organisations may challenge the land allocations, the lack of tenure security in existing informal settlements, housing density, and availability of PHP subsidy finance, they do not challenge the housing subsidy programme itself. However, inadequacies in the housing programme as well as more fundamental governance challenges appear to lead to considerable frustration on the part of social movement organisations as they see limited progress in addressing housing need. The following Section reports on the strategies used by the two social movement organisations seeking to improve access to shelter for their members. As we suggest below, present tensions may be symptomatic of the inability of the policy and the programme to respond adequately to the situation.

## 6 Strategies of social movement organisations

This section reports on the strategies used by FedUP and AbM as explained by interviewees from the organisations. The discussion then reports on the understanding of all interviewees with respect to the nature and success of these strategies.

### 6.1 FedUP strategies

The struggles of the South African Homeless People's Federation and the subsequent Federation of the Urban Poor (FedUP) to engage with and hence transform state housing programmes are already longstanding. A key tool in their mobilisation is the practice of daily savings. Patrick Magebula, President of FedUP and resident in Piesang River, Durban, explains: “the savings concept [in FedUP] is where people save as a collective – they take and use the money as a collective. It is not for individual needs but for community group needs.” Through savings, low-income residents, mainly women, are brought together in their neighbourhoods to create collectives that are able to challenge the power relations that create and maintain their disadvantage. As the members, mainly women, save together, they consider their development needs and how to tackle the problems they face in their daily lives. Savings provides a financial asset but, more importantly, a collective resource to address immediate and longer-term needs. Savings groups from different settlements link together through local exchange programmes and groups are encouraged to federate to be a political entity able to negotiate with the city. Groups are also encouraged to develop their own solutions to the problems that they face. Community-designed approaches for housing development are both recognised to be more cost effective, and contribute to broader goals as they capacitate local communities with skills and expertise. A core organising slogan of the Federation in South Africa is ‘Power is Money and Knowledge’. As a federation, savings scheme members seek to negotiate with local, provincial and national authorities, looking for ways to secure resources and support for their plans.

Reflecting the core needs of their constituency, land and housing are priority areas for collective action by FedUP. However, Patrick Magebula emphasised that this is only a part of what is needed: “our main issue is poverty – and we use land and housing as an entry point.” Despite this statement, most of the activities focus on housing. With respect to the needs of landless members, multiple land acquisition strategies (land purchase, negotiations with state agencies able to allocate land, and land invasion) have been followed. State housing subsidies are used to finance associated residential development. Once land is secured, savings schemes are encouraged to develop their own settlement layouts and housing designs with self-build approaches to construction. As explained in Section 3, an intensive programme of lobbying and negotiation helped to secure the People's Housing Process as a sub-programme within the capital subsidy (Baumann, 2003). When asked about strategies, leaders explained that the organisation is following a 24 point plan with land occupation as a final option.^21^ However in practice much of their work is executed through partnering with the state for housing construction. In 2006, FedUP secured national support for an allocation of 9000 subsidies (Sisulu, 2006). This finance is delivered through the provincial governments as these are the agencies responsible for disbursing housing subsidies.

Activities in eThekwini initially centred on Piesang River, a peri-urban settlement home to one of FedUP's national leaders, and then extended to include other settlements including Newlands West and Cato Crest. Relations with local government were strained during the late 1990s when the municipality attempted the eviction of Federation members from a park following their displacement due to internal violence within their settlement. A further incident took place in 1999 in the district of Lamontville where four houses were constructed by Federation members. The municipality demolished these dwellings under the Prevention of Illegal and Unlawful Occupation Act. Following a march by Federation members, the municipality rebuilt these four houses but refused to allow further development. Notwithstanding the difficulties faced by particular groups, 2735 houses have been constructed by FedUP members in eThekwini using state subsidy finance of which more than 900 units are in Piesang River.

Much of FedUP's construction in eThekwini took place between 1996 and 2000. Interviewees explained that the more stringent regulatory context is now delaying construction (although once plans are approved the subsidy finance is made available). A second reason is that the Federation and its support NGOs used to pre-finance housing subsidies through the community-managed fund, uTshani (which was partly capitalised by national government). However, difficulties in securing the release of subsidies have prevented the continuation and expansion of this programme (see Baumann & Bolnick, 2001 for an elaboration of these problems). FedUP has sought a closer alignment with the state (at national, provincial and municipal levels) to reduce delays in access to subsidy finance. As noted above, in 2006 they secured a commitment from the national Minister to facilitate access to housing subsidies (Sisulu, 2006) (although this partnership was rarely mentioned in interviews with Federation members) and some years before that, in 2003, they reached a formal partnership with the city authorities in eThekwini. Despite such relationships, Federation members explained that progress in housing construction is very slow.

FedUP's current primary strategy is to negotiate access to the housing subsidy programme. In 2008 one frustrated group invaded land but they were rapidly removed by the municipality. FedUP seek to identify objectives and activities of common interest with the government. Between 2003 and 2007, a staff member of uTshani was jointly selected and seconded to the municipal government with responsibility for facilitating the partnership with FedUP. However the city did not agree to continue this arrangement and in 2007 the individual began working as a support professional with FedUP. Monthly meetings with municipal staff continue and the municipal has promised a number of sites, however members are still waiting for exact locations and the beginning of substantive development. The leaders say that they have sought to work with Abahlali baseMjondolo but that little progress had been made.^22^

### 6.2 Abahlali baseMjondolo strategies

Abahlali baseMjondolo has grown out of a long-established local organisation in Kennedy Road, close to the centre of eThekwini, where some 2600 families are living.^23^ This settlement is located close to a municipal dumpsite. The community had long anticipated securing additional land near to their existing site so as to reduce over-crowding and improve the quality of housing. However, the geology of the area led the municipality to declare the site unsuitable for residential occupation.^24^ In February 2005, land located close to the settlement which the community believed had been promised to them was cleared for commercial use and the residents organised a road blockade in protest. S’bu Zikode, President of AbM reflected back on this incident and explained that “We did not know anything about organising demonstrations then, we did not know much about politics. The random blockade was illegal. Fourteen of us, we were arrested. We were very angry and frustrated.” From that date, activities grew and in 2009 the movement organisation had affiliates in 34 informal settlements in the province (most located in eThekwini) (see 12 above).

A central objective of their campaign is to enable communities in informal settlements to secure tenure of the land that they presently occupy and access subsidies to improve their homes.^25^ They believe that the policy context is favourable but that the municipality is not complying with the existing legal framework. In their view, the municipality is acting aggressively to remove citizens from their homes to redevelop informal settlements. S’bu Zikode explained that they have challenged many evictions of shack dwellers in the courts and only one has been legal. He explained that: “We are accused of just fighting government but we were formed to partner not to fight… We began to use the law as a sword but before we used it as a shield (when we were arrested). We came to understand that the law is balanced, if you have resources to use then it can help you.”

In addition to legal challenges, AbM have used three further tactics. First, demonstrations have expressed their frustration about the lack of housing. They had the expectation that they would be listened to by the state but relations rapidly became confrontational. S’bu Zikode suggests that the success of this tactic has been because of the violent reaction of the state. “We organised marches, very legal. But they ignored us. Then they were very stupid. In beating people they exposed themselves. They gave us more space and publicity. Their attitude changed with international and national shaming. On 28th September, we had a march, it was very well coordinated and complied with the Gatherings Act. By now we understood what was required by the Act and we complied. The church leaders were in the forefront. They used water cannons. This march opened a window for us, there was a lot of pressure condemning the beatings.” The leaders used famous personalities to attract media attention and submitted a video to the South African Human Rights Commission (SAHRC) to expose their living conditions.

A second tactic has been to improve access to basic services through illegal connections to water and electricity services. S’bu Zikoke elaborated on the results: “At first the municipality came to disconnect but then we reconnected. Eventually they stopped coming to disconnect. At one time, officials were stoned. Now, even if you have a legal connection and ask them to come and repair it, they will come but they will ignore the illegal part.” A third and less well-used tactic was developed in March 2006, when the organisation encouraged its members to boycott the local government elections under the slogan ‘No Land, No House, No Vote’. Members explained that despite the boycott they did not vote for an alternative political party and the ANC won the ward election. There is a consensus inside and outside of Kennedy Road that residents’ support the ANC; however, they did not want to be taken for granted. This experience of protest combining with party loyalty resonates with the findings of Matlala and Bénit-Gbaffou (2012) and research on social movement activism in Phiri.

AbM considered the Elimination and Prevention of the Re-emergence of Slums Act to be hostile to their members’ interests and those of other informal settlement dwellers. As a result of their initial protests prior to the passing of the Act, the KZN legislature came to their centre and debated with the community. However, members felt that they struggled to have their voices heard within this forum. As a result, they have challenged this Act in the courts.

Following their march in 2007, AbM recognised that the city government began to show greater interest in working with them and a local service NGO, the Project Preparation Trust (PPT), was appointed to mediate and assist in the preparation of a joint local authority and AbM plan to address the most essential needs. Trust staff worked with AbM to develop 14 pilot upgrading projects (to provide basic services) in settlements in which members were active and three areas for full subsidy-financed redevelopment. This Plan was concluded at the end of March 2009, and at the time of interviews the Kennedy Road community were awaiting the delivery of the programme. Municipal officials explained to us that the upgrading provision is similar to that offered to other informal settlements receiving temporary relief. Subsequent to this, AbM leaders were driven from Kennedy Road.

AbM did not begin its work with the support of a NGO and for considerable periods it has not had regular access to external finance to support its activities. It has also lacked access to professional advice in respect of housing construction and other aspects of settlement development. The relationship that has developed with Church Land Programme is supportive but does not offer housing expertise.

### 6.3 Perspectives on strategies – collaboration and contestation

The two movement organisations in Durban have pursued multiple strategies to secure tenure and housing finance. FedUP has moved from a mixed strategy including both confrontation and attempted collaboration in the late 1990s to a partnership with the city. They have secured limited access to land and subsidies supported by their relationship with the Department of Housing. AbM, established in 2005, found its early protests met with police force and had a confrontational relationship with the municipality until late in 2007. Negotiations began early in 2008 and by 2009 leaders had signed a joint plan with municipal officials and were awaiting implementation. Officials recognised that relations had been difficult with AbM but suggested that when offered the opportunity to negotiate, AbM responded positively because they appreciated the significance of some form of collaboration. It is also evident that movement positions are not singular even at one moment in time. One local group may be seeking to contest, while another is more conciliatory: and both may be supported by the leadership of the organisation. A discussion may be broadly collaborative while, in some cases, legal measures are being pursued and/or a more critical written position taken. Movement strategies appear both complex and dynamic. A number of arguments emerge to explain significant shifts by the government towards and away from outcomes that are viewed by the movement leaders as progressive, and to explain why movements change their strategies to enhance their influence.

As evidenced above and as is consistent with the literature, engagement with the state is considered to be critical by those seeking to address housing need in eThekwini.^26^ The strategies of FedUP and AbM are considered by many interviewees to be on opposite ends of a continuum of contestation and collaboration with the state. However, this binary distinction appears simplistic. From the organisational histories discussed above, FedUP and its predecessor, the South African Homeless People's Federation, have contested the policies of the municipality while AbM has been negotiating with the authorities. Such variation in strategies is consistent with the discussion of the literature in Section 4 which suggests it is important to go beyond contentious politics to understand the engagement of social movement and the state. This sub-section discusses collaborative engagement and confrontation in turn.

FedUP members evidenced what they had achieved by citing their record of housing construction, the institutionalisation of their relationship with the municipality, regular working group meetings with provincial and national government, and on-going housing developments on 400 plots. Support for a strategy of collaboration is given by other interviewees from NGOs. A staff member at the Project Preparation Trust argues that there is a need for practical interventions that demonstrate different approaches. He suggested that groups need to bridge the gap between frustrated citizens and the state in situations “where contestation is an easy option” but which does not of itself bring progress. Reflecting on his own work, he summarised his experience: “[B]y engaging with the people, they [state and civil society] come up with solutions. People come down the road and toyi toyi as they have never had access. Now [they] refocus their energies at the policy level.” As community organisations gain in understanding and capacity, they become more willing to explore options and reach compromise.

However, FedUP members themselves are frustrated at the slow speed of progress and some are questioning the effectiveness of their strategy. Access to land has been secured, but there is a feeling that relatively little has been achieved for the effort that has been made. Two challenges are widely recognised, the first of which is among the generally accepted problems discussed in Section 5. FedUP's activists believe that the bureaucracy is resistant to their demands for community-led development and that officials are discomforted by community control of housing projects. One of FedUP NGO support workers argued that it is: “…[v]ery hard to get technical staff in the municipality to help – [they] don’t want to deal with the federation.” One official suggested that FedUP has had limited significance to date because of state technical processes and government's “instruments and tools have not been aligned” with the principles of participation within the broader policy. FedUP members resist some state regulations (in part because they have observed an increase in regulations without evident commensurate benefits), although they have sought to improve internal capacity to comply with the regulatory framework.^27^ Similar struggles with state bureaucracy were also mentioned by staff from the Built Environment Support Group and the Church Land Programme drawing on their own experiences. With respect to the wider movement literature, Mohanty et al. (2011, 27) refer to the growing process of managerialism in eThekwini with limited opportunities for participation in state processes (ibid, 27), and their conclusion is supported by this research with the technical approaches of the state acting to constrain the engagement of movement organisations and local residents in housing development. As a consequence of the delays, municipal officials are dubious about the ability of the People's Housing Process (PHP) to deliver at scale. As one official argued “We are under a lot of pressure to deliver and PHP takes a long time.” One more senior official concluded “value depends on delivery at scale” while another critiqued the PHP because “[it] is not going to clear the slums” due to its slow pace. The official responsible for informal settlements explained that the municipality seeks to develop larger areas because of scale efficiencies. With this emphasis, there is little space for community participation and inclusive decision-making.

The second major challenge preventing FedUP's strategy of collaboration is that local councillors (and some community pressure groups) are suspicious of community-led development. This challenge is mentioned both by civil society commentators, professionals and community members working with FedUP, and local authority staff. To advance projects, councillors need to support the work; for example, the Community Liaison Officer in Cato Crest, when asked about the possibility of a local FedUP savings scheme doing their own construction within the municipal-led redevelopment explained that: “We do have that group and it is possible to fit them in. They have to knock on the doors of those above them.” He elaborated that by this he meant both local councillors and established community leaders. Another municipal staff member explained that “Councillors and pressure groups fight to control the projects” while other officials elaborated on the tendency of councillors to view local community groups as a potential political opposition. One ex-councillor concluded that, in his view, “it was hard for communities to manage councillors” and that “some councillors see FedUP as a threat.” One independent professional shared his view that FedUP was not able to challenge the power of local elites, and there is some evidence that AbM and indeed the council officials also face a similar problem.^28^ While councillors may not influence the national policy, they do exert a powerful influence over who is included in local developments.

Both of these challenges highlight the issue of power and control within the housing programme and produce a picture of a multi-layered process with contesting ideologies and interests. It appears that the success with which local community groups manage their relations with different groups within the state is central to the achievement of improved housing options. FedUP (and others) successfully negotiate the higher echelons of the state and secure support but believe that they are blocked by those lower down the decision-making hierarchy. Contrasting positions taken by different levels of the state are also evident in Thorn and Oldfield's (2011) account of the struggles of one community to resist a threatened eviction in Cape Town. In this case, FedUP are struggling to identify an effective response and are frustrated by slow progress. To pressure the state, FedUP members explained to us how they seek to invite the council to events whereby they can demonstrate the scale of their membership, with the incipient threat that mass mobilisation implies.

While this analysis is focussed on collaboration as a strategy of social movements, it is important to note that several state officials identified functional benefits for government from collaborating with groups such as FedUP. For government officials, working with FedUP helps both to improve the housing product and to create a more collaborative practice of policy development between the state and civil society. A (middle-level) official exemplified the first of these benefits when he explained that “I think it [PHP] has merits. It is very community-driven. This is good thing. You build your house, and you will take care of it even more, rather than someone coming in and giving you a key. What you notice [with contractor housing] is that when a tile comes off, then some say government come and take care of it but it doesn’t happen. There is no sense of pride and ownership. When you put your sweat and tears into it – then there is a sense of ownership.” Such perspectives suggest that there is a genuine interest in working together. A challenge for the social movement organisation is to build support for this understanding and then to use this support to advance its cause.

Turning to strategies of contestation, the experience of both FedUP and AbM is that (while there have been differences in the scale and intensity of opposition) an initial period of public confrontation has been followed by a more conciliatory engagement with the state. Does this point to the success of contestation or its limitations? Or does it just point to the limitations of extracting strategies for analysis outside an understanding of their historical context? A challenge is to avoid getting caught up in specific events but to look beyond specificities to understand what they tell us about the underlying and on-going processes, and the iterative interaction between outcomes and opportunities.

While the progression of AbM from contestation to negotiation is noted by all interviewees, there is little agreement about the underlying reasons for the change. A general lack of consensus on this process is exemplified by attribution of responsibility for the commencement of negotiations on the AbM plan and the involvement of the Project Preparation Trust (PPT). One city official and the President of AbM explained that the Plan was commissioned by the City who identified PPT to support the process. Another city official suggested that the PPT was involved because AbM wanted an independent mediator and when the City proposed PPT as a possible candidate, AbM agreed. Meanwhile a PPT staff member stressed that although they were contacted by the city to ascertain their willingness to assist, “it was Abahlali who approached us since we have a working relationship with the city”. Our point is not to find the “truth” in these accounts but to recognise that there are multiple perspectives on these events; it appears from the explanations that such multiple perspectives are an important part of the acceptability of specific activities.

AbM activists consider that due to their strategies and activities the organisation has achieved both a change in the discourse related to shack developments in eThekwini and specific commitments for the development of their own settlement. Negotiations with the municipality to provide limited services in 14 informal settlements neighbourhoods with the full subsidy-financed upgrading in three settlements carried on over several months with the MOU finally being signed in February 2009. While some resettlement would be required in Kennedy Road, the process of development was agreed. Movement activists believe that the violent reaction of the government to the march in September 2007 led to a change in the government's position and is responsible for the government's willingness to prioritise the development of AbM-affiliated settlements. In explaining the reasons which led to the Plan, AbM's President argued that “The city, it has had enough of us. It cannot afford people making this noise.” FedUP members and professional associates concur with this understanding that the municipality has responded to AbM's strategy of contention and also see the willingness of the state to negotiate as being a consequence of AbM's emphasis on public campaigns and willingness to contest the lack of housing provision “on the streets.”

An alternative perspective is offered by some interviewees from government agencies, and several officials suggested that the government was willing to negotiate before the march in September 2007. They argued that negotiations began late in 2007 because of a changed position of AbM and their willingness to talk (whereas previously they ignored overtures by the authorities), together with a change in their professional intermediaries. One senior official explain why AbM was ready to negotiate:

[W]hat also made a difference is that in their ward other communities moved into houses with proper facilities. Things were happening in other places, and in Kennedy Road and Foreman Road nothing was happening. Abahlali realised that they may be living in an island. They tried to get it [housing] through the international media but this was not going anywhere…

Although some officials argue that there is no clear link AbM protests and negotiations – some concede that campaigning does provoke a response from the municipality. “No, there was no link with the march in September 2007” argued one senior official “but Kennedy Road did get some preference. They received a lot of attention and we responded.”

### 6.4 Conclusion

The primary focus of the social movement organisations considered here is to improve housing through securing access to the subsidy. The problems with the lack of scale of the programme at the city level are rarely raised although problems with the processes related to subsidy acquisition and use are tabled. The social movement organisations work towards addressing the first two of the housing problems discussed in Section 5 above. The implicit assumption is that the current processes will be able to accommodate all of the members of both AbM and FedUP although of course this is not possible. Increased access will be associated with growing membership and the contradiction will be exposed.

Contestation and contention emerge as part of the same process, entwined strategies that position and reposition movement organisations in their attempts to extract resources from the state, improve the regulatory context and gain greater social recognition and status. Alternating strategies illustrate the reality that housing improvements are not a simple claim or entitlement that can be achieved (or not) by a campaign to influence a single decision. Rather such improvements are a complex intervention in people's lives that requires negotiation around a myriad of separate decisions made at multiple levels within government. The desire to secure the subsidy encourages collaboration as some level of negotiation and agreement is required. The shifts between collaboration and contestation reflect the both scale of resources available and the desire of state and civil society to access and control such resources. At one moment, movements may be trying to collaborate but the state may contest to advantage personal or political interests; at another time, these positions are reversed.

While collaboration and contestation is centred on asset acquisition, underlying this positioning are issues of both reputation and legitimacy. The theme of recognition and legitimacy resonates with a deeper challenge. Movement leaders acknowledge that while explicitly they are seeking a redistribution of assets (i.e. subsidy acquisition), their activities are important for other reasons and particularly the desire of those living in informal settlements to be treated as equal citizens and for such residency not to be the reason for discrimination and social and political exclusion. Being recognised as legitimate citizens making a contribution to urban prosperity and with a right to be in the city is seen as fundamental to their well-being. Being treated better is both an end in itself and a means to enhance a negotiating position to secure housing improvements and other assets and to avoid being marginalised. In addition to issues of respect for individual households and informal settlement dwellers, is the issue of organisational reputation and whether or not the social movement organisation is a ‘legitimate’ agency. As noted above, the first phase of the research emphasised that the theme of reputation and legitimacy is important in understanding which organisations are seen by the state as entitled to be involved in government programmes to address poverty and disadvantage.

This following section explores what this contributes to our understanding of the broader literature about both social movement strategies and housing policy in South Africa.

## 7 Conclusion

We consider here what the findings of this research mean in the context of issues raised in the literature discussing both social movements and housing in South Africa. We focus on four issues. The first is what we found out about the nature of the state and the relations between the housing social movements and the state. The second is the significance of professional support to social movement organisations. The third is related to the methodology of researching these topics and the fourth and final issue is what this means in terms of housing policy and addressing housing need. In terms of housing debates, the policy discourse and actual practice there are both immediate opportunities and constraints that need to be addressed if movements are to address the needs and interests of their members. These influence what can be achieved in the short-term, and the vision and ambition of movement organisations. Hence our study has enabled us to reflect back on the theoretical approach discussed in Section 4 and this is taken up in the first two sections below.

### 7.1 Social movement relations with the state

Compared to many countries, housing policy in South Africa is progressive. There is the availability of monies for housing subsidies, and the specific presence of the People's Housing Process sub-programme to facilitate greater community involvement. In eThekwini, this potential is augmented by an interest in engaging civil society from senior and some junior staff in both the municipality and the province. These individuals acknowledge that the state alone cannot address housing need, and other agencies have valuable ideas and activities to contribute. This view welcomes new ideas and alternative perspectives with one official suggesting “we need a difference of opinion to improve what is happening in this country.” Community organisations are seen as being able to address some negative consequences of the subsidy-driven context such as the culture of entitlement.

However, housing policies and programmes are not universally supportive of the involvement of either citizens or movement organisations. State officials in middle management are particularly concerned that community members comply with what is acknowledged to be an increasingly rigorous and complex regulatory framework. For these managers, established laws, rules and regulations rather than negotiations create the context within which movement activists have to further their goals. FedUP, with its decentralised self-managed constituency, struggles to deal with state officials that have little willingness to recognise the capacity and potential of its local members. As FedUP members contest the detail of delivery, much of their energy and momentum is taken up with interactions that deal with the technical delivery of housing. In this case, organisations are disadvantaged both because community members lack the skills and capacities, and because resistance is eroded as the nature of foundations, walls and roofs is checked, corrected, and rechecked. Dealing with such constraints has sharpened organisational capabilities but substantive difficulties remain.

The broader political context is also constraining. As recognised by Robins (2008), local councillors in South Africa are establishing clientelist relations, relations are highly personalised and challenges to the local power brokers may result in exclusion from benefits. Unlike the situation found in many countries, this is not a party political contest, and consistent with Matlala and Bénit-Gbaffou's (2012) research in Phiri, there is agreement from interviewees that support for the ANC remains strong. Rather the struggle appears to be related to councillors wishing to maintain their position, with housing subsidy finance being a resource through which this can be achieved. Traditional structures also vest power in local settlement leaders who influence the allocation of benefits for material gain and to be able to maintain power. The importance of clientelist politics has long been acknowledged. While Robins (2008) argues that South African movement organisations manage these politics with some success, Cherry et al. (2000, 901) suggest that relations may be difficult in the case of housing subsidy developments due to the scale of resources. The findings here suggest that there are substantive difficulties.

As summarised above, the literature recognises that social movement organisations use both contentious politics and more collaborative relations to created and open political opportunities to advance their cause. Ballard et al. (2006) suggest that in practice multiple strategies are followed (ibid, 404 and 406) and the findings above support this with both FedUP and AbM using a mix of strategies to advance their interests. Both organisations recognise the synergy between protest and engagement and while they take a different position on the continuum, both seek to be strategic in these choices. Mohanty et al. (ibid, 35) argue that collaboration comes at a cost and that “the closer to the state the social movement gets, the less likely it will be to achieve major transformatory changes; co-optation is more likely, if not inevitable.” But movement organisations in this study seek an engagement with government, trying to negotiate to advance their interests including access to the housing subsidy. While the state is resistant to activism and social protest and officials ostensibly stand back from and explicitly resist movement organisations that organise public protests, the evidence suggests such actions do influence their responses and that while such response does provoke distance by the state, this is accompanied by a desire to manage the protest through a negotiated accommodation in relation to subsidy finance.

In this context, the subsidy offers an opportunity for both resource acquisition and engagement with the state. Movements use the opportunity of the subsidy for mobilisation, and protest creates further opportunity, i.e. in the way described by social movement theorists whose work is summarised in Section 4. When movements use people's housing need as a source of grievance and political protest, it can be argued that they use the opportunity of the subsidy to strengthen their mobilisation. The government, through providing a housing entitlement, may be considered to be sanctioning some level of claim-making in this area. Subsidy acquisition is contested as illustrated in the case of eThekwini. Social movement organisations have, through the creation of the People's Housing Process, sought a subsidy delivery mechanism that favours both their material needs and their potential for strengthening local organisations. However, as shown above, the Process is of limited value in the context of limited access and increasing managerial controls. Movement organisations find that the state seeks to control this source of state redistribution. In this case, we can understand the programme in all its manifestations as a political opportunity, even if it is not simply one constructed by movements themselves. The findings that relate to programme establishment and re-definition are broadly similar to the discussion about social movement strategies and state responses in the global North (see Section 4 above).

The use of the subsidy opportunity for both collaboration and contestation challenges the emphasis on contentious politics within Tilly and Tarrow's articulation. It suggests a more complex framing needs to be in place which recognises the need for a multiplicity of strategies. Tarrow (1998) suggests that even apolitical movements may be conflictual, and here we see that politicised movements may also chose to avoid conflict if they believe there are more effective strategies to engage the state and advance their interests. Hence our findings are supportive of Goldstone's (2004) earlier conclusions. The findings here point to a genuine dilemma facing movement organisations that recognise that the use of contention is not simply to fight but to engage the state on more favourable terrain through a repositioning of the relations with the state. And that a challenge in the use of contention is to avoid de-legitimation both with the state and more broadly within political and civil society. As discussed above, historical practice influences modalities of action but it is also evident is that it is not enough to repeat such practice and it has to be adapted to be effective. A conclusion also suggested by Bénit-Gbaffou and Piper (2012, 175). The evidence suggests that repeating the forms of protest associated with struggles that ended almost two decades ago may no longer be successful in securing reputation and legitimacy.

The literature discussed in Section 4 includes a variety of views on the significance of invited participatory “spaces” and the significance of recognition and redistribution in the engagement of movements with the state. Both Thompson and Tapscott (2011, 16) and Bénit-Gbaffou and Piper (2012, 175) are critical of the potential of invited spaces. Once again our findings point to greater complexity. As shown in the discussion about the People's Housing Process, in this case participation in the People's Housing Process is itself a way to secure land and resources and realised access to housing. However it is important to note that the Process exists, at least in part, because the movement organisations have themselves supported such an investment. Hence this space is a hybrid lying somewhere between the dichotomy of invited and invented spaces. Piper and von Lieres (2011) quote Cornwell and Coelho (2007) to explain that invited spaces can, over time, become invented spaces. As the example shows here, the invented spaces of community-led housing construction became invited spaces within the People's Housing Process; these spaces were first positive as the state provided resources and then became more ambiguous as the state strengthened its regulatory control over state-financed construction. As shown here, such a space offers a potential but that potential is difficult to realise in full. Perhaps because of its hybrid nature, it is a contested space. In terms of the literature on invited and invented spaces, this point once again to the fallacy of simple binary distinctions. The presence of such spaces is seen by social movement organisations as a political opportunity through which to engage the state around an agenda that is their own and in so doing it assists in the formation of political relationships. It also legitimises the movement to a significant extent, thereby strengthening their political position. It provides an example of the ways in which movements have created openings which are then formed and reformed by state and movement interactions.

The emphasis has to lie in the dynamics – immediate outcomes are important but they cannot be considered outside of a consideration about what this means for the outcomes in the medium and longer-time. In this sense, our findings reiterate those discussed in Section 4: political opportunities are flexible and iterative. Moreover the findings here suggest that the processes involved are finely grained, nuanced and complex. For example, there is not a single set of interests within the state and as explained above local political groups may be threatened by engagements that higher levels of the state see as supportive to their objectives. A major challenge facing movement organisations in eThekwini is that, whatever the policy and programme advantages that are open to them, they need to deal with multiple levels within the state (senior, middle ranking and junior officials, and councillors) and this means that gains may be partial in their nature. What is notable is that state officials recognise the bureaucratic (regulatory) and the participatory modalities of state operation but appear to be much more ambivalent about the operation of clientelism. While many commentators acknowledge that it exists when questioned, they do not volunteer this information.

In this context, it is evident that movement organisations make political opportunities both by conflict and by consensus building. The finding is important because it highlights that the task is both to create political opportunities and to use opportunities, and both may require such skills such as technical expertise and less tangible assets such as legitimacy and a public reputation. These less tangible assets are important influences on the structure within which political opportunities are secured. Equally important is the broader political framework: for example, the ability of the authorities in eThekwini to manage housing resources and the constraints they face when operating a national programme. However, we are aware that this conclusion is somewhat general, and we return to this limitation after a discussion of relations in the follow sub-section.

Our findings substantiate and extend the earlier conclusion that in South Africa goals of both redistribution (‘old’ social movements) and recognition (‘new’ social movements) are important (see Section 4). Exclusion from both resources and control over resources is, in the minds of the shack dwellers, linked to negative perceptions of their identity as people living in informal settlements. Or, put another way, they recognise that inequalities in resource distribution are in part possible because of negative views and associated identities ascribed to those who are disadvantaged. Part of their interest in collaboration is to demonstrate their social worth, avoid marginalisation, promote a positive self-identity, reduce prejudice against the residents of informal settlements and so legitimate themselves and their organisations (Mitlin, 2013).

### 7.2 Relations within the movement

The ability to work across different groups within the state bears on one theme within the social movement literature which is the relationship between social movement organisations and social movement support organisations (Thompson & Tapscott, 2011, 10). Robins (2008, 167) argues that relations with professional agencies are important to the advancement of social movement struggles in South Africa as they facilitate more complex political strategies. Robins’ conclusions are reinforced in this study by the ways in which NGO staff, community city leaders and local residents engage with multiple levels of the state and negotiate agreements. AbM was considered by interviewees to be disadvantaged because it had not had the consistent support of a NGO with urban development skills and experience. As a result, it has had limited access to state officials. Lower and middle-level bureaucrats appear reluctant to respond positively to local community groups but may be willing to discuss potential collaboration with professionals and established organisations.^29^ Perhaps because of the absence of such linkages with officials, the members of AbM found their protests rapidly resulted in violent confrontational situations with state authorities. While the engagement with the Project Preparation Trust assisted in specific negotiations, this was not considered to offer the same benefits as a long-term relationship. Piper and von Lieres (2011) also emphasise the importance of the contribution of mediators (including the potential for them to add expert knowledge), in a context in which governance options are complex and marginalised communities have few social relations with the state. They argue that such mediators need not come from civil society. However, in this context, no-one suggested any other possibilities and it appear unlikely either that the individuals associated with the state could play this role, or that such mediators would emerge from the community itself. Also relevant here is the significance of international support (Thompson & Tapscott, 2011, 15); in part because it is considered to contribute to strengthening positive perceptions about the legitimacy of movement activities.

The relationships between movement organisations themselves does not emerge strongly in the recent literature on social movements in South Africa and there is a surprising lack of literature on the ways in which local groups compete against each other in Southern towns and cities and so weaken their potential to influence political outcomes.^30^ Such relations are recognised as a potential ‘opportunity’ in the social movement literature discussed above in Section 4, and they were discussed at some length by interviewees in this study. In the context of our research, respondents recognised the need for a unified voice representing the interests and needs of shack dwellers. However, interviewees explained how the municipality's actions, intentional or not, have exacerbated tensions between the movement organisations (and hence reduced the likelihood of collaboration). One professional working with AbM explained how AbM felt that FedUP was being held up as the ‘good guys’ and used to suggest to AbM that there was no need for them to form an alternative movement organisation. From his perspective, “…it was the ANC which created the tension between AbM and SDI (not ever FedUP though) by criminalising and repressing AbM and telling them that if they wished to avoid arrest they must affiliate to SDI.” Municipal staff explained that they tried to persuade AbM to work with FedUP (although not necessarily proposing membership) because they had an established relationship with FedUP. An alternative view was expressed by a professional working with FedUP explained how the new municipal Plan for AbM had resulted in FedUP feeling ‘bypassed’, with members feeling that they have had scant reward for their patient and continued willingness to persuade ambivalent bureaucrats and politicians of the value of community-led approaches.

Three different perspectives emerged from interviewees with respect to the relationship between these two social movement organisations. Some NGO support professionals perceive that the movements are in competition and argue that the alternative ‘counter’ view of the other movement organisation should be contested. While the benefits of cross-movement collaboration are recognised, this recognition is accompanied by demands as to what one movement organisation should require from the other prior to collaboration. A second perspective held by some NGO professionals, movement organisation leaders and members is that the movements have common interests, and that they both need each other and should collaborate. As argued by one civil society activist who has worked with both organisations, on the one hand, FedUP need to be able to critique the local authority, and on the other AbM need to learn how to collaborate. A third perspective, held by some movement organisation members, is that while the movements may not have a strategic interest in collaboration, they share a common identity and should not be criticised to outsiders, including those undertaking this study. While there have been some joint meetings between the two movement organisations in 2009 (and the minutes were shared with us), there also continue to be tensions within the relationships. Greater collaboration between movement organisations is, more generally, supported by Ballard et al. (ibid, 404) who recognise that contestation over strategies and broader goals between reformist and radical movements may be misplaced as “both sides actually require each other.” In this sense, the lack of collaboration may be considered to be a missed opportunity to strengthen and so advance claims for negotiation and access to the subsidy.

These findings emphasise that, rather than any static consideration of opportunities, movement organisations and their members recognise and respond to a dynamic and shifting terrain that they negotiate with openings that are in part being determined by their own agency including their capacity to create relationships. In this conclusion, the findings support the work of Goodwin et al. (1999). Their strategies seek to challenge ideas that reduce recognition of their citizenship as well as pursue policy and programme change. Their activities may be confrontational or may be concerned with presenting new information to the state and other influential agencies and individuals. In the case of FedUP, for example, emphasis is placed on savings activities in part because this is successful in changing the attitudes of the state and helping officials and politicians to see the residents of informal settlements in a positive light (Mitlin, 2013). Political opportunities have been created and improved by the strength of the local organisations (see Bebbington, 1999 and Appadurai, 2001 for further examples of this) and both organisations sought to strengthen their own capabilities so as to use existing opportunities to better effect. As organisations maintain their organising practice, so they better understand strategic actions in any given context. Capabilities in managing a triad of relationships, within the movement, between movement organisations (both horizontal and vertical), and engaging with the state are critical, as is the skill set associated with achieving legitimacy and recognition. As noted above, changing social attitudes towards the urban poor is of primary concern both to advance the demands of the social movement organisations, and because of the discrimination faced by their members. This capacity is linked to the ability to manage contestation and collaboration. As suggested by Goodwin et al. (1999, 53) movement organisations sharpen their capabilities as they reflect on and adjust to the strategies of the state.

As concluded in the section above, there is evidence to suggest that political opportunities are important, and that relations with professional groups and other movement organisations constitute such opportunities. However, considering both this conclusion and the analysis earlier in this section the emphasis on opportunities does not seem helpful beyond this level of generality. The opportunities that matter self-evidently vary in specific localities, and are also likely to vary across sectors. For example, realising improved housing in South Africa depends in part on the historically influenced meaning attributed to housing as well as the government's response to housing need as understood shortly after democratisation in 1994. Moreover while political opportunities are influenced by structural issues, these structures are not fixed; the discussion above shows, for example, how historical practices of protests, government policy and the legitimacy of movement actions are all in flux. Political opportunities discussed here have, in part, been defined by the campaigns of movement organisations including in this case the creation of a specific sub-programme. However, such opportunities have also been defined by the failings of others, notably the poor quality housing provided by commercial developers and the increasingly rigid specification related to housing provision. Moreover, while attention should be focussed on opportunities and structural change, it is also critical to think about what capabilities are needed to understand and respond to such opportunities. In summary, there are few generalities that are helpful in the attempt to understand specific struggles and their outcomes.

### 7.3 Researching movements

In terms of the methodology, Mohanty et al. (2011, 10) suggest that the approach of political opportunity structures enables us to work out “exactly what dynamics are taking place”. This study suggests that it can be more difficult than that. As shown by the different perceptions around the participation of Project Preparation Trust, there are many different views and multiple rationalities for such divergence. Moreover the ebbs and flows in these relationships remind us of the danger of “snapshots” which assess strengths, weaknesses and outcomes at a particular point in time. It is the deeper underlying trends that are more significant to our appraisal of the strategies used and the success they achieve. Particular activities and alliances will shift according to the nature of the moment, and their significance may only be apparent some distance into the future. We argue that it is difficult to understand the dynamics that are taking place and they are often only evident once they have taken place and been assessed. This does not mean analysis is impossible but it does suggest that it has to be undertaken cautiously. The danger, as suggested in an early analysis of the FedUP's precursor the South African Homeless People's Federation, is that while our understanding of events may be more focussed with the advantage of time, it reconstructs a simplified understanding of what actually took place (Bolnick, 1993). The trajectory of change, including the influencing of political opportunity, may be clear when looked back on from some future point, but when it is being negotiated it remains volatile and uncertain and capable of following multiple trajectories.

### 7.4 The social movement contribution to addressing housing need

What have we learned about the significance of the shelter social movement in eThekwini to low-income and disadvantaged people who are inadequately housed?

The broad context in South Africa is one of a considerable state commitment to address housing need as evidenced by both the scale of the programme and the size of each unit subsidy. Since 1995, housing policy has focussed on a capital subsidy for housing with units being allocated to entitled households in new-build developments. Programme amendments have including an increasing unit value of the subsidy to enable improvements in the quality of provision. Since 2004, housing policy has recognised the need to augment this programme with specific measures to upgrade existing informal settlements with greater emphasis on the improvement of living conditions for residents currently located in these areas; but until recently there have been few upgrading initiatives. The policy direction for housing suggests confidence in state delivery. At the general level, a number of criticisms have been made about the housing subsidy programme that subsequent amendments have sought to overcome (Section 2). The city of eThekwini has been the location through which to explore the interface of social movement organisations with housing delivery. As with other cities in South Africa, low-income African, Indian and Coloured families struggled to remain close to the city centre in Durban during the years of apartheid government. Some residents of informal settlements managed this, although with considerable efforts. There are also many thousands of families living in more peripheral settlements with inadequate access to basic services, uncertain tenure and very poor-quality housing. There is an active set of civil society organisations working on these issues including grassroots groups, social movement organisations and NGOs.

Robins (2008, 172), Swilling (2008, 508) and Miraftab (2003) are all broadly optimistic about what can be achieved by social movement organisations struggling for improved shelter in South Africa. Robins argues that the groups in Cape Town associated with the South African Homeless People's Federation were able to manage both patronage politics and the technocratic state relations to their advantage (ibid, 83 and 96). Success seems more difficult in eThekwini. However, this difference in findings appears to reflect the different spatial scale of the research. Robins (2008) is focussed on a particular greenfield settlement whereas the focus here has been on the city. Swilling (2008) and Miraftab (2003) are discussing things from a city or national perspective. For grassroots groups seeking neighbourhood-level improvements at scale in eThekwini, some progress has been made in terms of accessing resources within specific settlements, but considerable needs remain as described above. While the formal commitment to participation is in place and even augmented by additional processes, its intent appears thwarted by multiple objectives including an orientation to high standards and formally managed construction and hence the conclusions in Section 3 in relation to a paucity of participation in projects are supported. The members of both social movement organisations believe that their needs and interests are, at best, only partially taken into account in subsidy-financed housing developments and that they have been too few occasions on which they have been able to negotiate access to an adequate number of housing subsidies.

In terms of both shelter-related issues facing the urban poor in eThekwini and the housing programme currently being used to address them, there was a high degree of consensus among interviewees with a primary focus on three areas of concern. All three areas reflect the national-level debates. Interviewees mentioned the high degree of regulatory control, and the adverse consequences of such regulation. Second, there was recognition of the problems associated with limited access to well-located land, the frequent need to relocate some residents when dense informal settlements are upgraded, and the lack of well-serviced well-located land for those for whom relocation is required. Third, there were concerns about the inadequate scale of the programme as subsidy-related construction appears not to be keeping pace with in-migration; as a consequence, informal solutions continue to be important. All three of these problems correspond to the concerns identified in the literature (Section 3).

Despite agreement on the challenges, in terms of the way forward opinions are mixed. Both the state and the movement organisations viewed securing housing only in terms of access to the housing subsidy although both recognised the inadequate scale. The movement organisations have sought access for their members partly on their own terms and partly through compromise, and both movement organisations believe they have had some success in addressing members’ housing needs. AbM have secured the right for at least some of their members to remain on their present site (local authority officials were seeking to remove all residents due to environmental health concerns) and have an outline plan for a subsidy-financed upgrading in the medium- to long-term with an understanding that some residents will have to be relocated. FedUP members have built over 2500 houses in the province (primarily in eThekwini and mainly in the late 1990s), and are making some progress with their current negotiations. Relative to other residents in housing need, members of the social movements have received several benefits. In particular, they appear to be able to accelerate development processes and they are more likely to gain a measure of autonomy within the upgrading and development process enabling greater value to be generated through the development. However, it may also be argued that the gains secured by movement organisations are small when set against their considerable efforts, their members’ needs and the 200,000 plus households waiting for shelter improvements in the city. While gains have been secured, movement organisations have struggled to make substantive progress commensurate to the scale of need. Moreover, the limited ability of the municipality to respond to what are viewed by low-income residents and their organisations as legitimate needs and demands has led to a consciousness among low-income residents that they are being denied dignity and respect.

Despite shortcomings in government policy and programmatic direction, present movement struggles are, as Ballard et al. (2006, 402) argue, located in members’ present realities and hardships and seek to engage the state through the government's expressed interest in addressing poverty. Hence activities aim to access the subsidy with a number of strategies to respond to problems that block the redistribution of resources to local residents. The relatively high unit value of the housing subsidy may help to explain the orientation of movement organisations to a particular approach to housing problems. Potential access is a powerful influence on the movement process and, although there appears to be little prospect of a majority of those in need being assisted in the short- and medium-term, little emphasis is placed on alternatives to the subsidy and more substantive change. Mohanty et al. (2011, 25) reach a similar conclusion when they comment on the lack of attention to systemic change in respect of social movement organisations’ work towards participatory local governance. Equally, housing officials also acknowledge some of the structural weaknesses in the policy and that important delivery issues are not being addressed, but nevertheless continue with the current direction. The recent Elimination and Prevention of the Re-emergence of Slums Act can be seen as one political (and penal) response to concerns about how the city authorities can keep pace with housing need. However, as explained above, there is no possibility that controls alone can prevent people moving to urban areas, at least not the kind of controls that the constitution would find acceptable. Moreover if informal settlement expansion is controlled but alternatives are not provided, then households will continue to living in inadequate and unsafe shelter (albeit it increasingly in formal settlements). Their inability to conceptualise and realise alternatives relevant to their own context emphasises the concerns of Heller and Evans (2010) raised in Section 4: urban policies are controlled by the national discourse and associated programming.

Both movement organisations and the state appear to be constrained by the present policy framework, struggling to define choices outside of the boundaries that this imposes. As noted by Khan and Pieterse (2006, 158–159), the leadership of the South African Homeless People's Federation recognised that the state might not deliver development but continued to believe in a constructive engagement with the state. Arguably, it is only when the politics progresses to the point where the debate is how ‘to house the city’ is it likely that the nature of the discourse and associated policy and programme and response will change. Greater collaboration between the two movement organisations would help to highlight the inability of the present approach to address the problem. If each organisation is focussed on its members, then the scale of solutions that are sought is more likely to be confined. Raising the level of the debate will mean reversing the current thinking with its focus on the individual dwelling and the size of the programme, and thinking instead about the numbers in need. Such a direction is likely to require taking upgrading more seriously, densification and will almost certainly involve re-thinking the use of subsidy finance. And since this research took place, the policy at the national level is shifting in that direction. At the same time, for this process to be realised, it will also require a much more conscious engagement with collective organisations because of their potential contribution to effective and efficient housing improvements, something the existing policy with its emphasis on the individual household has failed to address.

## Funding source

Economic and Social Research Council ESRC grant number, RES-16725-0170.

## Conflict of interest

There may be a perceived conflict of interest in that I (Diana Mitlin) work part-time for the University of Manchester, and part-time for another organisation, the International Institute for Environment and Development which is one of the funders of the international network to which one of movement organisations researched is affiliated (Shack/Slum Dwellers International). I have not been involved in any funding decisions related to SDI affiliates, including the SDI affiliate in South Africa. I have not worked closely with the South African affiliate since 2004 when we completed a joint research project.

## Footnotes

1 Many of these goods and services are public goods and individual supply is inappropriate. In the case of land and housing, these are goods whose provision is managed (at least in theory) by the state within a regulatory framework. While land is not a public good in this sense, the scale of provision (and hence the costs) is heavily influenced by state involvement in land zoning, land development and infrastructure investments.

2 Smith, D. (2008, July 27). Zuma's political honeymoon slips into a winter of discontent, *Guardian*, http://www.guardian.co.uk/world/2009/jul/27/letter-from-africa-jacob-zuma (downloaded: Saturday, 05.09.2009). The previous day, the Observer had reported: “Across South Africa's 283 municipalities, similar incidents have caused a crescendo of rage in the past month. Protesters have brandished placards saying life was better under white rule.” Duval-Smith, A. (2009). Zuma plea as protests sweep the townships, *The Observer*, http://www.guardian.co.uk/world/2009/jul/26/jacob-zuma-township-rally-kwazulunatal (downloaded: Saturday, 05.09.2009).

3 The key principles which guide housing policy and strategy include: restoring and furthering human dignity and citizenship, maximum private sector involvement and a product which is demand led (DoH 2003, 3). However, note the conclusion of Huchzermeyer (2003a, 212) that this is an essentially “supply driven approach”.

4 www.housing.gov.za.

5 Dollar rand exchange rates have varied over the last 15 years but for most of the period they have been between 6 and 8 rand to the dollar. In this paper we use 7 rand to the dollar as a reasonable approximation. For more details of exchange rates, see www.imf.org.

6 A ‘top up’ amount was introduced for areas in which building costs were particularly expensive including the Western Cape and KwaZulu Natal.

7 http://www.housing.gov.za/default.htm (media release, 15 December 2008, accessed Sunday, 15.02.09).

8 From Press Release From bare fields to the backyards of properties: the shifting pattern of informal dwelling erection, dated 24 November 2008, South African Institute of Race Relations, www.sairr.org.za, downloaded Thursday, 12 February 2009.

9 This included 105 representatives of civil society organisations, the majority of which were from CBOs.

10 Presentation of the City Manager to the Parliamentary Monitoring Group, 28 June 2011. Available at http://www.pmg.org.za/report/20110629-department-human-settlements-accredited-municipalities-nelson-mandlea (accessed 08.07.12).

11 http://www.witness.co.za/index.php?showcontent&global[_id]=20089 (downloaded May 16, 2009).

12 Mfusi. (2009). November 9th Slums Act now pointless, http://www.themercury.co.za/index.php?fArticleId=5236531. See, http://www.constitutionalcourt.org.za/site/abahlali.htm (downloaded, Tuesday, November 10, 2009).

13 See: http://www.wits.ac.za/academic/clm/law/cals/housingandevictions/11202/advocacy_and_media_releases.html.

14 Abahlali baseMjondolo means shack dwellers in Zulu language. Abahlali was interviewed in both phases one and two of the research project.

15 The number of 34 settlements was that mentioned in the interview with S’bu Zikode. Richard Pithouse (personal communication, 4th March 2009) elaborated that in November 2008 (the last date at which it was recorded) membership includes paid up individual membership (i.e. individuals holding a 2008 membership card): 10,000; settlements collectively affiliated to the movement: 15 (all in Durban); settlements with branches but not affiliated to the movement: 19 in Durban and 20 nationally (including Pietermaritzburg, Howick, eShowe and Cape Town).

16 As discussed in Section 2 above, there has been a significant strengthening of the empowerment approach, at least at the policy level, with Breaking New Ground (DOH, 2004). The interest of the first Minister of Housing following democratisation in providing initial capital of 10 million Rand to the uTshani Fund may reflect some recognition within government of the value of different approaches.

17 This number is thought by municipal staff to be close to a complete assessment. It is a considerable increase from the estimate of 108 (see above) and this reflects changing boundaries and new assessments.

18 The plots have to be at least 150 m^2^ with 80 m^2^ of build-able area.

19 Problems with quality observable in previous years are considered by all to have been resolved, perhaps because the municipality provides an additional R20–25,000 per plot for tarred roads and mains sewerage, a higher infrastructure specification than the national standards. Municipal staff explained that this is required for two reasons; the topography results in gravel roads being washed away and pit latrines are not appropriate in the high-density settlements commonly found in the city.

20 Municipal officials explained this was because of a change in national policy. Communities are only allowed to access an additional subsidy for an electricity connection once. It is most efficient to install these services when full upgrading takes place.

21 This “24 point plan” was agreed early in 1997 by the Federation leadership. The plan outlines a number of activities to strengthen savings schemes and prepare for rapid site development. Federation groups that wish to invade land are required to show that they have followed the steps outlined in the plan before receiving Federation support; invasion is seen considered to be acceptable if activities related to negotiation and compromise in securing land have been attempted and failed.

22 Since the interviews took place a city network including FedUP and AbM has been established.

23 www.abahlali.org/node/16, accessed Tuesday, December 30, 2008. See also Pithouse (2008b).

24 However it was pointed out to us by one academic that technical surveys from consultants contracted by the city indicate that significant portions of the land can be developed, and this is consistent with the willingness of the City to permit some development. It was also suggested that the local middle-class residents may be lobbying for the removal of the shack dwellers.

25 Although this remains an important demand, the Kennedy Road community now accept that not all of the existing families will remain on the site.

26 When asked about movement strategies at the scale of the city, interviewees failed to identify organisations other than FedUP and AbM. Individual groups at the settlement level were identified.

27 For example, at the national level they have developed Community Construction Management Teams to improve building quality in self-build development, and built relations with contracting companies such as Peer Africa and Urban Dynamics.

28 In September 2009, Abahlali leaders were attacked within Kennedy Road and driven from their homes. One interpretation of this violence is that local ANC leaders including councillors were threatened by the possibility that subsidy finance was about to be forthcoming for a development controlled by the community. See, for example, the discussion on www.abahlali.org and www.pambazuka.org.

29 As researchers, we experienced this directly through our interactions with members of the Siyanda KwaMashu Newlands Interface Housing Forum, a group concerned about their allocations in a housing development adjacent to their shacks. This group had been unable to secure a response from those responsible for the development but when we intervened the Department of Housing responded within a few weeks.

30 See Posner (2012) for a discussion of competition between local groups seeking to access housing subsidies in Chile.
